# CD11c identifies microbiota and EGR2‐dependent MHCII^+^ serous cavity macrophages with sexually dimorphic fate in mice

**DOI:** 10.1002/eji.202149756

**Published:** 2022-05-24

**Authors:** Calum C. Bain, Pieter A. Louwe, Nicholas J. Steers, Alberto Bravo‐Blas, Lizi M. Hegarty, Clare Pridans, Simon W.F. Milling, Andrew S. MacDonald, Dominik Rückerl, Stephen J. Jenkins

**Affiliations:** ^1^ Queens Medical Research Institute University of Edinburgh Centre for Inflammation Research Edinburgh UK; ^2^ Department of Medicine Columbia University New York New York USA; ^3^ Institute of Infection, Immunity, and Inflammation University of Glasgow Glasgow UK; ^4^ Simons Initiative for the Developing Brain, Centre for Discovery Brain Sciences University of Edinburgh Edinburgh UK; ^5^ Lydia Becker Institute for Immunology and Infection, School of Biological Sciences, Faculty of Biology, Medicine & Health University of Manchester Manchester UK

**Keywords:** macrophage ⋅ peritoneal cavity ⋅ regulation

## Abstract

The murine serous cavities contain a rare and enigmatic population of short‐lived F4/80^lo^MHCII^+^ macrophages but what regulates their development, survival, and fate is unclear. Here, we show that mature F4/80^lo^MHCII^+^ peritoneal macrophages arise after birth, but that this occurs largely independently of colonization by microbiota. Rather, microbiota specifically regulate development of a subpopulation of CD11c^+^ cells that express the immunoregulatory cytokine RELM‐α, are reliant on the transcription factor EGR2, and develop independently of the growth factor CSF1. Furthermore, we demonstrate that intrinsic expression of RELM‐α, a signature marker shared by CD11c^+^ and CD11c^–^ F4/80^lo^MHCII^+^ cavity macrophages, regulates survival and differentiation of these cells in the peritoneal cavity in a sex‐specific manner. Thus, we identify a previously unappreciated diversity in serous cavity F4/80^lo^MHCII^+^ macrophages that is regulated by microbiota, and describe a novel sex and site‐specific function for RELM‐α in regulating macrophage endurance that reveals the unique survival challenge presented to monocyte‐derived macrophages by the female peritoneal environment.

## Graphical abstract

Monocyte‐derived antigen presenting cells in the peritoneal cavity are heterogenous, with two discrete populations distinguished by expression of CD11c, dependence upon microbiota, CSF1R, and the transcription factor EGR2, and distinct sex‐dependent contribution to resident CD102^+^ macrophages. These results help us understand the diverse factors regulating monocyte fate in serous cavities.

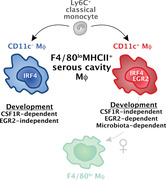

## Introduction

The serous cavities are home to two populations of macrophages which, in mice, can be defined by their differential expression of F4/80 [1, 2]. F4/80^hi^ macrophages dominate the cavities under normal physiological conditions and can also be distinguished by their high expression of the cell‐surface molecule CD102 (ICAM2) or the transcription factor GATA6 [3, 4, 5]. In contrast, their less abundant F4/80^lo^ MHCII^+^ coinhabitants rely on the transcription factor IRF4 and express around 130 genes that distinguish them from monocytes, F4/80^hi^ peritoneal macrophages, and other tissue macrophages, with CD226 (DNAM‐1), CD206, and RELM‐α among the most differentially expressed [6, 8]. Expression of CD226, RELM‐α, and CD206 also distinguish mature F4/80^lo^MHCII^+^ macrophages from conventional DC (cDC) found within cavity of CSF1R^+^ F4/80^lo^MHCII^+^ cells [6, 8–10]. F4/80^hi^ and F4/80^lo^MHCII^+^ cavity macrophages also display distinct turnover kinetics. F4/80^lo^MHCII^+^ macrophages are short‐lived and continually replenished by classical Ly6C^hi^ monocytes whereas F4/80^hi^ macrophages are relatively long‐lived, derive from embryonic precursors, and have the ability to self‐maintain [6, 7, 11–13]. However, embryo‐derived F4/80^hi^ macrophages are gradually displaced by BM‐derived cells with age [6, 14], but in a sex‐dependent manner [6, 15, 16]. Notably, BM‐derived F4/80^hi^ cells share numerous features of F4/80^lo^MHCII^+^ macrophages including expression of MHCII and RELM‐α and lack of TIM4 [6, 15]. While F4/80^lo^MHCII^+^ cells appear able to differentiate into F4/80^hi^ (MHCII^–^) cells in the absence of an intact resident population [17], the exact ontogenetical relationship between these populations remains unclear.

A key goal in the field is to understand the transcription factors and microenvironmental signals that govern macrophage differentiation in different tissues [18]. While the development and transcriptional signature of resident F4/80^hi^ peritoneal macrophages is controlled by the transcription factors C/EBPβ, RXR, and GATA6 [3–5, 17, 19], F4/80^lo^MHCII^+^ macrophages are dependent on the transcription factor IRF4 [7]. Development of F4/80^lo^MHCII^+^ cavity macrophages is also seemingly sensitive to prolonged treatment with combination broad spectrum antibiotics, which, has led to the idea that this compartment requires signals from microbiota to differentiate [7].

Several studies have subdivided F4/80^lo^MHCII^+^ peritoneal macrophages based on intensity of surface CD11c expression [6, 8, 9, 20]. The CD11c^+^ fraction exhibits greater proliferative activity [6], while the CD11c^–^ cells are larger [5], express higher levels of CSF1R and are more dependent on CSF1 for survival [20]. Despite differences in surface CD11c expression, both populations are labeled with YFP expression in CD11c^Cre^ *.Rosa26* ^LSL‐eYFP^ mice, suggesting that the CD11c^–^ cells may derive from the CD11c^+^ fraction [6]. However, the relevance of CD11c‐based partitioning of these cells is questioned by their shared expression of many other common macrophage and APCs receptors [9], equivalent capacity to capture [9, 20], process, and present antigen [8] and inability to activate naïve T cells [9], and because in some studies, CD226^+^ peritoneal macrophages appear to homogenously express high levels of surface CD11c [7].

Here, we show that while mature RELM‐α‐expressing F4/80^lo^MHCII^+^ peritoneal macrophages arise postnatally, only the CD11c‐expressing population do so in response to coloniation with the microbiota. We identify the transcription factor early growth response 2 (EGR2) as essential for the generation of mature RELMα^+^ CD11c^+^ F4/80^lo^MHCII^+^ peritoneal macrophages but largely dispensable for generation of their CD11c^–^ counterparts or F4/80^hi^ cavity macrophages. Using a combination of fate mapping approaches, we demonstrate that CD11c^+^ and CD11c^–^ F4/80^lo^MHCII^+^ macrophages appear to represent independent differentiation outcomes of monocytes in the cavity. Finally, we demonstrate that constitutive, cell‐intrinsic expression of RELM‐α regulates persistence of F4/80^lo^MHCII^+^ macrophages and BM‐derived F4/80^hi^ macrophages, albeit in a sex and cavity‐dependent manner.

## Results

### Microbiota drive emergence of a CD11c‐expressing subset of F4/80^lo^MHCII^+^ macrophages

Analysis of germfree mice has revealed that the microbiota or their derivatives control homeostasis of tissue macrophages in barrier tissues [21, 22] and distal sites including the brain [23]. As CD226‐expressing F4/80^lo^ MHCII^+^ macrophages are reported to arise after birth at a time when mice become colonized and since development of F4/80^lo^MHCII^+^ peritoneal macrophages is disrupted by antibiotic treatment [7], we set out to determine definitively the importance of microbiota in imprinting the F4/80^lo^MHCII^+^ peritoneal macrophage phenotype.

**Figure 1:**
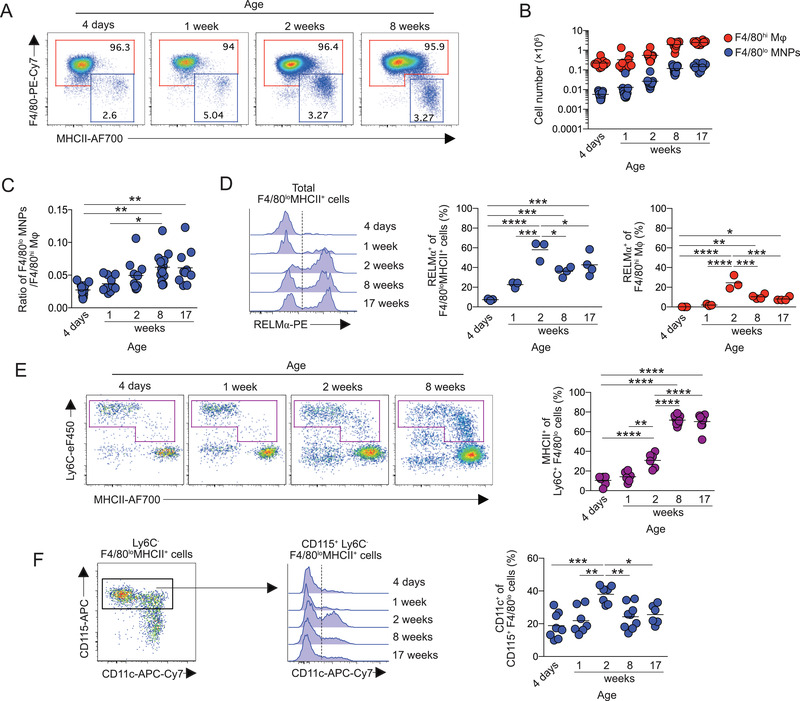
**Development of peritoneal macrophages after birth**. Peritoneal cells were isolated from mice of mixed sex at various ages between 4 days and 17 weeks and analyzed by flow cytometry (for full gating strategy, see Supporting information Fig. S1). (A) Representative expression of F4/80 and MHCII by live CD45^+^ Lin^–^ CD11b^+^ peritoneal cells. Data are from one experiment representative of three performed. (B) Number of F4/80^hi^ and F4/80^lo^MHCII^+^ peritoneal MNPs obtained at the indicated ages. Symbols represent individual animals with line at mean of 9–13 mice per group pooled from three independent experiments. (C) Ratio of F4/80^hi^ to F4/80^lo^ MHCII^+^ peritoneal macrophages from data in (B). (D) Representative expression of intracellular RELM‐α by total F4/80^lo^MHCII^+^ MNPs (*left*) and proportion of RELM‐α expressing F4/80^lo^ MHCII^+^ MNPs and F4/80^hi^ resident macrophages at the indicated ages. Symbols represent individual animals with line at mean of three to four mice per time point from one experiment. (E) Representative expression of Ly6C and MHCII by CD45^+^ Lin^–^ CD11b^+^ F4/80^lo^ cells (*left*) and proportion of Ly6C^+^ F4/80^lo^ cells that express MHCII (*right*) at the indicated ages. Symbols represent individual animals with line at mean of six to eight mice per group pooled from two independent experiments. (F) Representative expression of CD115 and CD11c by F4/80^lo^ MHCII^+^ MNPs (*upper*) and CD11c expression by CD115^+^ F4/80^lo^ MHCII^+^ MNPs (*lower*) at the indicated ages. *Right*, proportion of CD11c expressing cells among CD115^+^ F4/80^lo^ MHCII^+^ MNPs. Symbols represent individual animals with line at mean of six to eight mice per group pooled from two independent experiments. Symbols on graphs represent individual mice with an n of 9–13 (B), 3–4 (D), or 6–8 (E‐F) mice per time point pooled from 1 (D), 2 (E‐F), or 3 (B) experiments. *p < 0.05, **p < 0.01, ***p < 0.001, ****p < 0.0001 (C‐F; one‐way ANOVA).

We first verified changes that occur in the peritoneal macrophage compartment after birth. Our analysis confirmed that F4/80^hi^ and F4/80^lo^MHCII^+^ mononuclear phagocytes (MNPs) were present from birth (Fig. 1A; Supporting information Fig. S1). However, although both populations increased in number as mice aged (Fig. 1B), the ratio of F4/80^lo^MHCII^+^ cells to F4/80^hi^ macrophages also increased with age, suggesting the relative abundance of F4/80^lo^MHCII^+^ cells increase until adulthood (Fig. 1C). Expression of RELM‐α, a signature molecule of F4/80^lo^MHCII^+^ peritoneal macrophages in the adult [6], was absent from these cells in the neonatal period and was gained progressively with age (Fig. 1D), similar to that described for CD226 expression by these cells [7]. Other phenotypic changes also occurred in the F4/80^lo^ compartment during this time. In particular, differentiation of Ly6C^hi^ monocytes through the so‐called monocyte “waterfall” appeared to be absent in neonatal mice, whereas Ly6C^+^MHCII^+^ monocyte‐macrophage intermediaries could be detected among the Ly6C^+^ fraction from 2 weeks of age (Fig. 1E). CD11c^–^ and CD11c^+^ subpopulations were detected among the mature CD115^+^ Ly6C^–^ F4/80^lo^MHCII^+^ macrophage fraction at all ages, although their abundance peaked at the onset of monocyte differentiation through the monocyte waterfall, before reducing in number in adulthood (Fig. 1F). A small proportion of F4/80^hi^ peritoneal macrophages also express RELM‐α, corresponding to those cells most recently derived from BM [6, 15]. Expression of RELM‐α in F4/80^hi^ cells also only occurred postnatally (Fig. 1D). Thus, RELM‐α expression in F4/80^lo^MHCII^+^ and F4/80^hi^ macrophages occurs after birth and parallels the onset of monocyte differentiation through an MHCII^+^ state in the peritoneal cavity.

**Figure 2:**
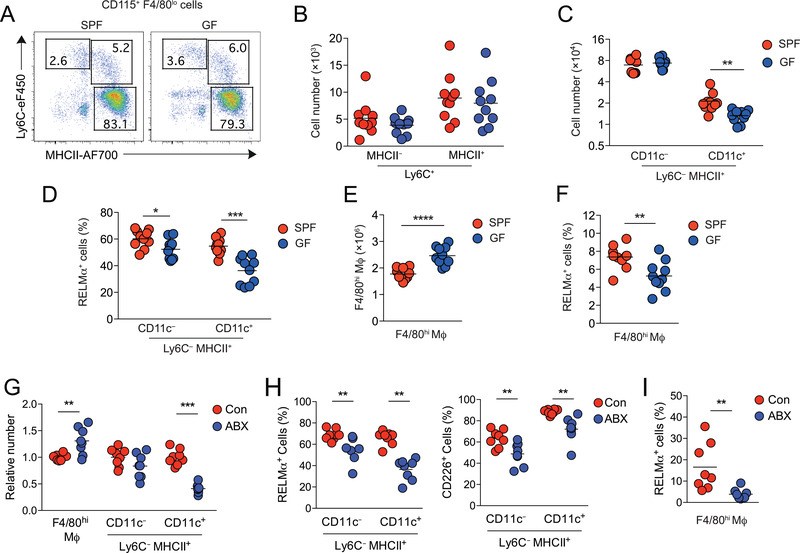
**Effects of microbial colonization on peritoneal macrophages**. (A) Representative Ly6C and MHCII expression by live Lin^–^ CD11b^+^ CD115^+^ F4/80^lo^ peritoneal leukocytes obtained from 6‐ to 9‐week‐old SPF or GF male mice. (B) Absolute number of Ly6C^+^ MHCII^–^ and Ly6C^+^ MHCII^+^ cells in the peritoneal cavity of SPF and GF mice detailed in (A). (C) Absolute number of CD11c^–^ and CD11c^+^ fractions of Ly6C^–^ MHCII^+^ CD115^+^ F4/80^lo^ macrophages in the peritoneal cavity of SPF and GF mice detailed in (A). (D)Frequency of RELM‐α^+^ cells within CD11c^–^ and CD11c^+^ fractions of Ly6C^–^MHCII^+^CD115^+^F4/80^lo^ cells from the peritoneal cavity of SPF of GF mice detailed in (A). (E) Absolute number of F4/80^hi^ macrophages in the peritoneal cavity of SPF or GF mice detailed in (A). (F) Frequency of RELM‐α^+^ cells within F4/80^hi^ macrophages from the peritoneal cavity of SPF or GF mice detailed in (A). (G) Relative number of F4/80^hi^ macrophages and CD11c^–^ and CD11c^+^ fractions of Ly6C^–^ MHCII^+^ CD115^+^ F4/80^lo^ macrophages of male mice treated with an antibiotic “cocktail “of vancomycin, neomycin, ampicillin, and metronidazole (ABX) or without (control = Con) antibiotics normalized to numbers in controls. H. Frequency of RELM‐α^+^ (left) and CD226^+^ (right) cells within CD11c^–^ and CD11c^+^ fractions of Ly6C^–^MHCII^+^CD115^+^F4/80^lo^ cells from the peritoneal cavity of mice detailed in (G). (I) Frequency of RELM‐α^+^ cells within F4/80^hi^ macrophages from the peritoneal cavity of mice detailed in (G). Symbols on graphs represent individual mice with an n = 8 (ABX study) or 10 (GF study) animals per group pooled from two independent experiments. *p < 0.05, **p < 0.01, ***p < 0.001 (C, D, G, H, Student's *t*‐test with Holm–Sidak correction; E, F, I, Student's *t*‐test).

To determine definitively the role of commensal microbiota in induction and homeostasis of the F4/80^lo^ MHCII^+^ peritoneal macrophages, we next compared the peritoneal compartment of adult germ‐free (GF) mice with specific‐pathogen‐free (SPF) mice. The numbers of Ly6C^hi^MHCII^–^ monocytes and their Ly6C^+^MHCII^+^ descendants were comparable between SPF and GF mice, indicating that monocyte recruitment to the peritoneal cavity is independent of microbes and/or their products (Fig. 2A and B). We did find a small difference in the frequency and absolute number of F4/80^lo^MHCII^+^ Ly6C^–^ cells in GF mice compared with their SPF counterparts, but this effect was restricted to the CD11c^+^ fraction (Fig. 2C). GF conditions also led to a minor reduction in proportion of F4/80^lo^MHCII^+^ peritoneal macrophages expressing RELM‐α, an effect also more apparent in the CD11c^+^ fraction (Fig. 2D). Interestingly, the absolute number of F4/80^hi^ macrophages was routinely higher in GF than SPF mice (Fig. 2E), whereas the proportion expressing RELM‐α was reduced (Fig. 2F). Thus, microbial colonization is not the dominant factor driving emergence of mature F4/80^lo^ peritoneal macrophages or recruitment and maturation of Ly6C^hi^ monocytes after birth, but specifically drives the generation of a CD11c^+^ subset of these cells and the emergence of RELM‐α^+^ resident macrophages.

Given that the effect of microbiota on homeostasis of peritoneal macrophages was more subtle than reported for antibiotic treatment [7], we performed our own study on the effect of antibiotics. Consistent with previous studies [7], we found that prolonged treatment with broad‐spectrum antibiotics (ABX) led to a significant reduction in F4/80^lo^MHCII^+^ macrophages, but this was entirely restricted to the CD11c^+^ fraction of these cells, with relatively little effect on the number of CD11c^–^ F4/80^lo^MHCII^+^ macrophages or the proportion that expressed CD226 or RELM‐α (Fig. 2G and H). As in GF mice, there was also an overall increase in number of F4/80^hi^ resident macrophages, but a decrease in frequency of those expressing RELM‐α (Fig. 2G and I). Thus, both antibiotic‐treated and GF mice have a specific defect in peritoneal CD11c^+^ F4/80^lo^ macrophages and alterations in homeostasis of resident macrophages.

### F4/80^lo^ MHCII^+^ macrophages are largely equivalent between sexes

We and others have reported a significant effect of the sex of mice on the turnover, number, and transcriptional profile of F4/80^hi^ peritoneal macrophages [6, 15, 16, 24] and biological sex significantly effects the composition of microbiota that in turn drives sex dimorphisms in immune function [25, 26]. However, the total number of peritoneal cavity Ly6C^+^ monocytes and CD11c‐defined subsets of F4/80^lo^ MHCII^+^ macrophages, or the proportion expressing CD226 or RELM‐α was equivalent between mature male and female mice (Supporting information Fig. S2A–C). Furthermore, mRNA sequencing revealed that unlike F4/80^hi^ resident cells, F4/80^lo^ peritoneal macrophages from male and female mice were largely transcriptionally equivalent (Supporting information Fig. S2D–F; Table S1) and differed in only 2 of the 112 genes defined by others to comprise the unique transcriptional identity of these macrophages (Supporting information Fig. S2F) [7]. Hence, biological sex is not a major factor influencing frequency, composition, or transcriptional signature of F4/80^lo^ MHCII^+^ macrophages. We also compared C57BL6/J and C57BL/6N mice to determine if the variation in reported prevalence of CD11c^+^ cells within MHCII^+^ F4/80^lo^ peritoneal macrophages may be attributable to strain [6, 7, 8, 9], but despite marginal differences in cell numbers (Supporting information Fig. S3A) and levels of RELM‐α expression, the proportion of F4/80^lo^MHCII^+^ macrophages that expressed CD11c did not differ between substrains irrespective of sex (Supporting information Fig. S3A–C). Hence, neither sex nor C57BL/6 strain appears to affect the balance of the CD11c‐defined subsets of cavity F4/80^lo^MHCII^+^ macrophages.

### EGR2 expression is a selective property of F4/80^lo^ MHCII^+^ macrophages in the serous cavities

The transcription factor IRF4 has recently been identified to drive the development of CD226^+^ F4/80^lo^MHCII^+^ macrophages in the serous cavities [7]. However, because F4/80^lo^MHCII^+^ macrophages were reported to be uniformly CD11c^+^ in that study, it remains unclear if IRF4 controls development of both CD11c^+^ and CD11c^–^ subsets we reproducibly identify among the F4/80^lo^ MHCII^+^ compartment. Importantly, we found that IRF4 was uniformly expressed by all F4/80^lo^MHCII^+^ macrophages irrespective of CD11c or RELM‐α status (Supporting information Fig. S4A and B). Furthermore, analysis of CD11c^Cre^.*Irf4* ^fl/fl^ mice, in whom IRF4 was efficiently deleted from both CD11c^–^ and CD11c^+^ F4/80^lo^MHCII^+^ peritoneal macrophages and CD11b^+^ DC (Supporting information Fig. S4A and B) [7], revealed a loss of each of these populations (Supporting information Fig. 4C) and almost complete loss of RELMα expression in F4/80^lo^MHCII^+^ macrophages irrespective of CD11c subtype (Supporting information Fig. 4D). In contrast, F4/80^hi^ macrophages from CD11c^Cre^.*Irf4* ^fl/fl^ mice and *Irf4* ^fl/fl^ littermate controls were equivalent in number and RELM‐α expression (Supporting information Fig. 4E). Thus, IRF4 controls development or differentiation of all CD11b^+^ F4/80^lo^MHCII^+^ MNPs in the serous cavities.

**Figure 3:**
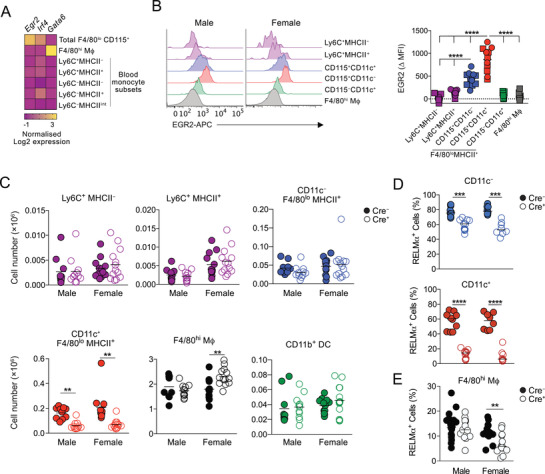
**EGR2 controls development of CD11c^+^ peritoneal F4/80^lo^MHCII^+^ macrophages**. (A) Heat map showing expression of *Egr2* and *Irf4* in the indicated peritoneal and blood populations from the ImmGen Consortium. The heat map displays the relative log2‐normalized expression per gene, calculated as the gene expression value of each cell type minus the mean expression value per gene over all cells in the heatmap. B. Representative expression of EGR2 by Ly6C^+^ MHCII^–^ and Ly6C^+^ MHCII^+^ monocytes, CD11c^–^ and CD11c^+^ fractions of Ly6C^–^ MHCII^+^ CD115^+^ F4/80^lo^ macrophages and F4/80^hi^ resident macrophages obtained from the peritoneal cavity of *Egr2* ^fl/fl^ mice (left) and mean fluorescence intensity of EGR2 expression in these populations from six male (square symbols) and five female (circles) *Egr2* ^fl/fl^ mice from one of three representative experiments. ****p < 0.0001 (one‐way ANOVA). (C) Absolute number of Ly6C^+^ MHCII^–^ and Ly6C^+^ MHCII^+^ monocytes, CD11c^–^ and CD11c^+^ fractions of Ly6C^–^ MHCII^+^ CD115^+^ F4/80^lo^ macrophages, F4/80^hi^ resident macrophages and CD11b^+^ DC in the peritoneal cavity of male and female *Lyz2* ^Cre^ *.Egr2* ^fl/fl^ (Cre^+^) and *Egr2* ^fl/fl^ (Cre^–^) mice. Symbols represent individual animals with 10 (male Cre^–^), 11 (male Cre^+^), 12 (female Cre^–^), and 13 (female Cre^+^) mice per group pooled from two to three independent experiments. **p < 0.01, ***p < 0.001 (Student's *t*‐test with Holm–Sidak correction). (D) Frequency of RELM‐α^+^ cells within CD11c^–^ and CD11c^+^ fractions of Ly6C^–^MHCII^+^CD115^+^F4/80^lo^ cells from the peritoneal cavity of Cre^+^ and Cre^–^ mice in (C). Symbols represent individual animals, with cells 10 (male Cre^–^), 11 (male Cre^+^), and 8 (female Cre^+^ and Cre^+^) mice per group pooled from two independent experiments. ***p < 0.001, ****p < 0.0001 (Student's *t*‐test with Holm–Sidak correction). (E) Frequency of RELM‐α^+^ cells within F4/80^hi^ peritoneal macrophages from Cre^+^ and Cre^–^ mice in (C). Symbols represent individual animals, with 13 (male and female Cre^+^), 15 (female Cre^–^), and 17 (male Cre^–^) mice per group pooled from four independent experiments. **p < 0.01 (Student's *t*‐test with Holm–Sidak correction).

Next, to identify additional transcription factors that may regulate development of these cells, we analyzed publicly available microarray data of peritoneal F4/80^lo^ and F4/80^hi^ macrophages together with all Ly6C and MHCII‐defined blood monocyte subsets (www.immgen.org) [6]. We found that *Egr2* was highly expressed by F4/80^lo^MHCII^+^ CD115^+^ cells compared with their F4/80^hi^ coinhabitants and blood monocytes (Fig. 3A and Supporting information Table S2). We confirmed this pattern of EGR2 expression at a protein level using flow cytometry (Fig. 3B). Importantly, EGR2 levels were higher and more uniform in the CD11c^+^ fraction of F4/80^lo^MHCII^+^ serous macrophages (Fig. 3B) and largely restricted to the mature RELM‐α^+^/CD226^+^ cells (Supporting information Fig. S5A and B). Expression of EGR2 was also not dependent upon sex (Fig. 3B & Supporting information Fig. S5C), and, unlike IRF4 (Supporting information Fig. S4A), it was not expressed by cavity cDC2 (Fig. 3B & Supporting information Fig. S5A–C). Hence, EGR2 expression largely defines the CD11c^+^ subset of F4/80^lo^ peritoneal cavity macrophages.

EGR2 is a member of the immediate early genes, a set of transcriptional regulators induced during transition from one cellular state to another. Although EGR2 has been implicated in monocyte to macrophage differentiation in vitro [27, 28, 29], its role in vivo remains unclear. To examine the requirement of EGR2 in the development of the F4/80^lo^MHCII^+^ subsets, we used *Lyz2* ^Cre^ *.Egr2* ^fl/fl^ mice which allow specific deletion of *Egr2* in all myeloid cells without the postnatal lethality seen in global *Egr2*‐deficient mice [30]. We confirmed that all monocytes and macrophages in the peritoneal cavity displayed high levels of Cre activity in this system by examining expression of tdTomato by peritoneal leukocytes from *Lyz2* ^Cre^.*Rosa26* ^LSL‐CAG‐tdTomato^ mice (Supporting information Fig. S6A). Blood monocyte subsets were present at equivalent frequencies in *Lyz2* ^Cre^.*Egr2* ^fl/fl^ mice (referred to here as Cre^+^) compared with their *Egr2* ^fl/fl^ littermate controls (referred to here as Cre^–^ mice) (Supporting information Fig. S6B and C). Similarly, the abundance of Ly6C^+^ monocyte subsets in the peritoneal cavity was unaffected by *Egr2* deficiency (Fig. 3C). In contrast, F4/80^lo^MHCII^+^ Ly6C^–^ cells in Cre^+^ mice were reduced in abundance compared with their Cre^–^ littermates, but consistent with the pattern of EGR2 expression, this was largely restricted to the CD11c^+^ subset (Fig. 3C). Notably, the loss of CD11c^+^ F4/80^lo^MHCII^+^ macrophages did not simply reflect a failure to upregulate CD11c, as we did not detect a reciprocal increase in the CD11c^–^ fraction (Fig. 3C) or the number of peritoneal CD11b^+^ DC affected by *Egr2* deficiency (Fig. 3C). Importantly, RELM‐α (Fig. 3D; Supporting information Fig. S6E) and CD226 expression (Supporting information Fig. S6F) by F4/80^lo^MHCII^+^ cells was reduced in the absence of *Erg2*, but this effect was much greater in the CD11c^+^ fraction despite a complete reduction in EGR2 expression in both populations (Supporting information Fig. S5D and F). Almost identical deficiencies were detected between male and female mice, although CD226 expression appeared more dependent on *Egr2* in females (Fig. 3C, Supporting information Fig. S6E). These data suggest EGR2 is largely dispensable for development of F4/80^lo^MHCII^+^ peritoneal macrophages per se, but plays a nonredundant role in the differentiation and/or survival of the CD11c^+^ subset. Consistent with their negligible expression of EGR2, there was no difference in the general phenotype of F4/80^hi^ macrophages in Cre^+^ mice compared with Cre^–^ littermates (Supporting information Fig. S6G). However, we found a modest increase in number of resident F4/80^hi^ peritoneal macrophages (Fig. 3C) and a reduction in the proportion that expressed RELM‐α^+^ in female Cre^+^ mice (Fig. 3E), despite no overlap in the expression of RELM‐α and EGR2 in these cells (Supporting information Fig. S5D), suggesting a potential sex‐dependent role for EGR2^+^ cells in regulation of resident macrophages and their replenishment from the BM.

### The relationship of CD11c^+^ and CD11c^–^ serous cavity macrophages

**Figure 4:**
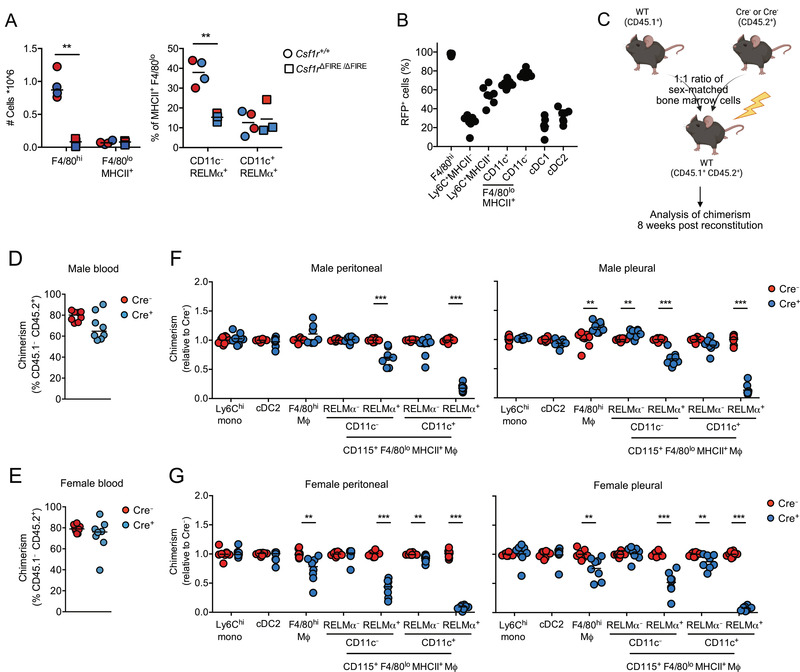
**Inter‐relationship of peritoneal macrophage subsets**. (A) Absolute number of peritoneal F4/80^hi^ and F4/80^lo^MHCII^+^ macrophages (left) and frequency of CD11c^–^ RELM‐α^+^ and CD11c^+^ RELM‐α^+^ cells within F4/80^lo^MHCII^+^ peritoneal macrophages (right) from *Csf1*r^+/+^ and *Csf1r* ^ΔFIRE/ΔFIRE^ mice. Symbols represent individual male (blue) or female (red) animals, with 4 *Csf1*r^+/+^ and 3 *Csf1r* ^ΔFIRE/ΔFIRE^ mice pooled from two independent experiments. **p < 0.01 (Student's *t*‐test with Holm–Sidak correction). (B) Frequency of RFP^+^ cells within the indicated cell populations from the peritoneal cavity of CD64^iCre/+^.*Rosa26* ^LSL‐RFP/+^ mice (both sexes). Symbols represent seven individual animals pooled from three independent experiments. *p* > 0.05 for all comparisons, except Ly6C^+^ MHCII^–^ versus CD11b^+^ DC that was not significant (one‐way ANOVA with Tukey's multiple comparisons test). (C) Experimental schematic for construction of mixed bone marrow chimeric mice. (D) Relative frequency of CD45.1^–^ CD45.2^+^ cells to CD45.1^+^ CD45.2^–^ cells within blood Ly6C^hi^ monocytes from male mixed bone marrow chimeric given bone marrow from WT CD45.1^–^ CD45.2^+^ and CD45.1^–^CD45.2^+^ bone marrow from *Lyz2* ^Cre^.*Egr2* ^fl/fl^ (Cre^+^) or *Egr2* ^fl/fl^ (Cre^–^) mice. Symbols represent individual mice. Data represent eight mice per group pooled from two independent experiments. (E) As in (D), but for female bone marrow chimeric mice. Data represent eight mice per group pooled from two independent experiments. (F) Contribution of CD45.1^–^ CD45.2^+^ *Egr2* ^fl/fl^ bone marrow to the indicated peritoneal and pleural cavity populations in male mixed chimeras given Cre^+^ or Cre^–^ bone marrow. Chimerism was normalized to Ly6C^hi^ blood monocytes before normalization of Cre^+^ to Cre^–^. Data represent eight mice per group pooled from two independent experiments. **p < 0.01, ***p < 0.001 (Student's *t*‐test with Holm–Sidak correction). (G) As in (F), but in female bone marrow chimeric mice. Data represent eight mice per group pooled from two independent experiments. **p < 0.01, ***p < 0.001 (Student's *t*‐test with Holm–Sidak correction).

We next examined the relationship between CD11c^+^ and CD11c^–^ serous cavity F4/80^lo^MHCII^+^ macrophages and their role as precursors of F4/80^hi^ resident cells. We recently demonstrated that CD11c^–^, but not CD11c^+^, F4/80^lo^MHCII^+^ peritoneal macrophages are rapidly depleted upon short‐term treatment with the CSF1R kinase inhibitor GW2580 [20], suggesting these may represent independent populations. To test this directly, we first used mice deficient in the Fms‐intronic regulatory element (FIRE) of the *Csf1r* gene, which lack the expression of CSF1R on F4/80^hi^ and F4/80^lo^ peritoneal macrophages [31]. As we previously reported [31], *Csf1r* ^ΔFIRE/ΔFIRE^ mice exhibited a marked reduction in F4/80^hi^ peritoneal macrophages yet retained normal numbers of total F4/80^lo^MHCII^+^ MNPs (Fig. 4A, left). Lack of CSF1R prevented division of F4/80^lo^MHCII^+^ MNPs into the subsets described above. However, by using RELM‐α^ ^as a proxy for F4/80^lo^MHCII^+^ macrophages, we could show that CD11c^–^ cells contributed almost threefold less to the MHCII^+^ population in *Csf1r* ^ΔFIRE/ΔFIRE^ mice, whereas CD11c^+^ cells remained unaffected (Fig. 4A, right) despite uniformly lacking of CSF1R expression (Supporting information Fig. S7A). Next, we used mice expressing Cre recombinase under control of the endogenous *Fcgr1* promoter [32] (referred to here as CD64^iCre^) since monocytes and CD11c^–^ F4/80^lo^MHCII^+^ macrophages but not CD11c^+^ F4/80^lo^MHCII^+^ macrophages express CD64 at the protein [6] and gene [9] level. CD64^iCre^ mice were crossed with *Rosa26* ^LSL‐RFP^ mice and the expression of red fluorescent protein (RFP) was assessed in the CD64^iCre/+^.*Rosa26* ^LSL‐RFP/+^ progeny. We found that peritoneal Ly6C^hi^ monocytes exhibited only low levels of labeling, but labeling increased as monocytes progressed through the differentiation “waterfall” (Fig. 4B). Importantly, CD11c^–^ F4/80^lo^MHCII^+^ macrophages showed significantly higher levels of RFP labeling than their CD11c^+^ counterparts, which were intermediate between Ly6C^+^MHCII^+^ monocytes and CD11c^–^ F4/80^lo^MHCII^+^ macrophages. Given that labeling in this system is irreversible, these data rule out the idea that CD11c^–^ cells are differentiation intermediates between monocytes and CD11c^+^ F4/80^lo^MHCII^+^ macrophages.

We originally proposed that all F4/80^lo^ peritoneal macrophages and monocyte‐derived F4/80^hi^ resident cells derive from a CD11c^+^ precursor due to the high level of YFP labeling observed in these populations isolated from CD11c^Cre^ *.Rosa26* ^LSL‐eYFP^ mice [6, 15]. However, the presence of largely normal numbers of CD11c^–^ F4/80^lo^ macrophages in *Lyz2* ^Cre^.*Egr2* ^fl/fl^ mice despite the almost complete absence of the mature CD11c^+^ F4/80^lo^ population suggests that CD11c^+^ cells may not be obligatory precursors of the CD11c^–^ fraction. To investigate this possibility further, we used competitive mixed BM chimeras generated from lethally irradiated CD45.1^+^ CD45.2^+^ WT mice reconstituted with a 1:1 mix of CD45.1^+^ WT and CD45.2^+^ Cre^–^ (*Egr2* ^fl/fl^) or Cre^+^ (*Lyz2* ^Cre^.*Egr2* ^fl/fl^) BM cells to assess the intrinsic dependence on *Egr2* for development of serous cavity macrophages from the BM (Fig. 4C). Eight weeks postreconstitution, *Egr2*‐deficient Cre^+^ BM was found to contribute as well as *Egr2*‐sufficient Cre^–^ BM to the generation of blood Ly6C^+^ monocytes (Fig. 4D and E) and peritoneal Ly6C^+^ monocytes, cDC2, and RELM‐α^–^ fractions of F4/80^lo^MHCII^+^ macrophages. In contrast, mature RELM‐α^+^ CD11c^+^ F4/80^lo^MHCII^+^ macrophages were completely dependent on EGR2 and derived almost exclusively from WT BM in WT:Cre^+^ recipients (Fig. 4F and G). Critically, mature RELM‐α^+^ CD11c^–^ F4/80^lo^ cells exhibited only partial dependence on intrinsic EGR2 expression (Fig. 4F and G), consistent with the minor reduction in number of these cells detected in intact *Lyz2* ^Cre^.*Egr2* ^fl/fl^ mice. Hence, these data strongly suggest that CD11c^–^ F4/80^lo^MHCII^+^ macrophages arise, at least in part, independently of their EGR2‐dependent CD11c^+^ counterparts.

This pattern in dependence of the short‐lived populations of peritoneal myeloid cells on EGR2 was largely similar across sexes, consistent with our findings in intact *Lyz2* ^Cre^.*Egr2* ^fl/fl^ mice, and was also mirrored by cells from the pleural cavity (Fig. 4F and G). Crucially, both RELM‐α^+^ CD11c^–^ and RELM‐α^+^ CD11c^+^ macrophages exhibited significantly greater dependence on EGR2 than F4/80^hi^ resident macrophages irrespective of sex or cavity site, suggesting neither population is a dominant requisite precursor of serous cavity F480^hi^ macrophages (Supporting information Fig. S7B and C). However, unlike in males (Fig. 4F), peritoneal and pleural F4/80^hi^ macrophages from female mixed WT:*Lyz2* ^Cre^.*Egr2* ^fl/fl^ chimeric mice exhibited a partial reliance on *Egr2* (Fig. 4G). This is consistent with the reduced frequency of RELM‐α^+^ F4/80^hi^ macrophages we had previously observed in only female intact *Lyz2* ^Cre^.*Egr2* ^fl/fl^ mice and suggests the overall increase in F4/80^hi^ peritoneal macrophages found in these animals does not arise through cell‐intrinsic negative regulation of F4/80^hi^ macrophages by EGR2 but from a cell‐extrinsic effect of *Egr2*‐deficiency in other *Lyz2*‐expressing cells.

Hence, together with our analysis of *Csf1r* ^ΔFIRE/ΔFIRE^, *Lyz2* ^Cre/+^.*Egr2* ^fl/fl^, CD11c^Cre/+^.*Irf4* ^fl/fl^, and CD64^iCre/+^.*Rosa26* ^LSL‐RFP/+^ mice, these data suggest that CD11c^+^ F4/80^lo^, CD11c^–^ F4/80^lo^, and F4/80^hi^ serous cavity cells represent largely independent macrophage populations, but with a limited sex‐dependent role for EGR2^+^ cells in replenishment of resident cavity macrophages from the BM.

### RELM‐α regulates peritoneal macrophage differentiation

**Figure 5:**
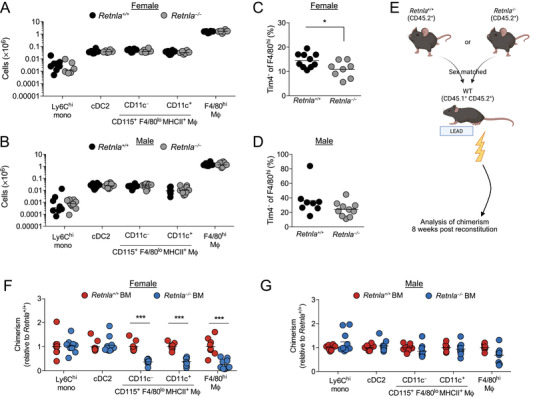
**RELM‐α promotes survival or differentiation of monocyte‐derived cells in the female peritoneal cavity**. (A) Absolute number of Ly6C^+^ MHCII^–^ and Ly6C^+^ MHCII^+^ monocytes, CD11c^–^ and CD11c^+^ fractions of Ly6C^–^ MHCII^+^ CD115^+^ F4/80^lo^ macrophages, F4/80^hi^ resident macrophages, and CD11b^+^ DC in the peritoneal cavity of female *Retnla* ^+/+^ and *Retnla* ^–/–^ mice. Symbols represent individual animals, with eight (*Retnla* ^+/+^) and six (*Retnla* ^–/–^) mice per group, pooled from three independent experiments. (B) As for (A) but from male *Retnla* ^+/+^ and *Retnla* ^–/–^ mice. Symbols represent individual mice, with eight (*Retnla* ^+/+^) or ten (*Retnla* ^–/–^) mice per group pooled from three experiments. (C) Frequency of TIM4^–^ cells within peritoneal F4/80^hi^ macrophages from mice in (A). Symbols represent individual mice, with ten (*Retnla* ^+/+^) or eight (*Retnla* ^–/–^) mice per group pooled from four experiments. (D) Frequency of TIM4^–^ cells within peritoneal F4/80^hi^ macrophages from male *Retnla* ^+/+^ and *Retnla* ^–/–^ mice in (B). (E) Experimental schematic for construction of tissue‐protected single bone marrow‐chimeric mice. (F) Contribution of CD45.1^–^ CD45.2^+^ bone marrow to the indicated peritoneal populations in female tissue‐protected chimeras given *Retnla* ^+/+^ or *Retnla* ^–/–^ bone marrow. Chimerism was normalized to Ly6C^hi^ blood monocytes before normalization to chimerism in mice receiving *Retnla* ^+/+^ bone marrow. Data represent nine (*Retnla* ^+/+^) or ten (*Retnla* ^–/–^) mice per group pooled from two independent experiments, except for Ly6C^hi^ monocytes in *Retnla* ^–/–^ group, where n = 9 as too few of these cells in one animal. ***p < 0.001 (Student's *t*‐test with Holm–Sidak correction). (G) As for (F), but in male tissue‐protected bone marrow‐chimeric mice. Data represent eight (*Retnla* ^+/+^) or nine (*Retnla* ^–/–^) mice per group pooled from two independent experiments.

Finally, we set out to determine the significance of the constitutive production of RELM‐α by peritoneal F4/80^lo^MHCII^+^ macrophages. RELM‐α is a pleotropic cytokine with both pro‐ and anti‐inflammatory function dependent upon context [33], yet its role in homeostasis remains unclear. Despite the high level of constitutive RELM‐α expression by F4/80^lo^MHCII^+^ macrophages, these cells were present in equal numbers in *Retnla* ^–/–^ mice compared with their *Retnla* ^+/+^ littermate controls irrespective of sex, as were F4/80^hi^ resident macrophages, CD11b^+^ DC, and Ly6C^+^ monocytes (Fig. 5A and B). However, the proportion of F4/80^hi^ resident macrophages with a TIM4^–^ phenotype, akin to the monocyte‐related RELM‐α^+^ cells [6, 15], was lower in RELMα‐deficient mice, although only in females (Fig. 5C and D), suggesting RELM‐α might regulate differentiation of monocytes into resident peritoneal macrophages in a sex‐specific manner. Hence, to assess the role of cell‐intrinsic expression of RELM‐α in differentiation and survival of macrophages, we generated tissue‐protected BM chimeric mice in which WT (CD45.1^+^CD45.2^+^) mice received irradiation to the hind legs, followed by reconstitution with sex‐matched *Retnla* ^+/+^ or *Retnla* ^–/–^ (CD45.2^+/+^) BM (Fig. 5E). Equivalent frequencies of donor‐derived cells were observed in circulating Ly6C^+^ monocytes 8 weeks after reconstitution irrespective of donor BM genotype (Supporting information Fig. S8A and B). Similarly, Ly6C^+^ monocytes and CD11b^+^ DC within the peritoneal cavity of both male and female chimeras derived equally from *Retnla* ^+/+^ or *Retnla* ^–/–^ BM, consistent with the lack of RELM‐α expression by these cells (Fig. 5F and G). In contrast, the chimerism of both CD11c^–^ and CD11c^+^ F4/80^lo^MHCII^+^ macrophages was lower for recipients of *Retnla* ^–/–^ BM but only in female chimeric mice (Fig. 5F and G) revealing an unexpected sex‐dependent role for RELM‐α in survival or differentiation of peritoneal F4/80^lo^ macrophages. There was also a striking reduction in the level of chimerism in F4/80^hi^ macrophages in female recipients of *Retnla* ^–/–^ BM, whereas this effect was less apparent in males. This dimorphism occurred even though replenishment of F4/80^hi^ macrophages from BM occurred overall at a much lower rate in females (Supporting information Fig. S8C and D), as we have previously reported [6, 15]. Notably, a female‐biased competitive advantage of RELM‐α expression was also observed in pleural F4/80^hi^ macrophages (Supporting information Fig. S8E and F), although this appeared less marked than in the peritoneal cavity and pleural F4/80^lo^MHCII^+^ macrophages exhibited no dependence on RELM‐α irrespective of sex. Hence, cell‐intrinsic production of RELM‐α contributes to maturation and/or survival of BM‐derived peritoneal F4/80^lo^MHCII^+^ and F4/80^hi^ macrophages in a sex‐ and site‐dependent manner and this reliance likely contributes to the altered phenotype of resident peritoneal macrophages obtained from female *Retnla* ^–/–^ mice.

## Discussion

Here, we demonstrate that the murine F4/80^lo^MHCII^+^ macrophage compartment comprises two distinctly regulated subsets demarked by CD11c expression. We reveal a nonredundant role for the transcription factor EGR2 in the development of CD11c^+^ F4/80^lo^MHCII^+^ macrophages and show that this subset is uniquely dependent upon the microbiota and independent of CSF1. Furthermore, we show that CD11c^+^ and CD11c^–^ F4/80^lo^MHCII^+^ macrophages develop largely independently and neither acts as an obligate nor dominant precursor of resident F4/80^hi^ peritoneal macrophages.

Our findings that the microbiota specifically regulates CD11c^+^ F4/80^lo^MHCII^+^ macrophages potentially explains why the prevalence of this subset changes during development and varies so greatly between studies. Specifically, differences in microbiota between animal units could explain why Kim and colleagues found all F4/80^lo^MHCII^+^ macrophages express CD11c and why they observed a greater than threefold loss of F4/80^lo^MHCII^+^ macrophages upon ABX treatment [7]. In our hands, CD11c^+^ cells generally comprised around a third of F4/80^lo^MHCII^+^ macrophages in adult mice and as a result, GF and ABX‐treated animals had only marginally fewer total F4/80^lo^MHCII^+^ macrophages. Notably, we used mice from six different animal units across three universities, including one non‐SPF facility, yet in none was the CD11c^+^ fraction dominant. Hence, colonization by commensal microbiota is not the dominant factor driving emergence of peritoneal F4/80^lo^MHCII^+^ macrophages after birth but rather dictates the generation of the CD11c^+^ subset of these cells.

Despite broad similarities in gene expression, phagocytic capacity and antigen presentation of [8, 9, 20] CD11c^–^ and CD11c^+^ F4/80^lo^MHCII^+^ macrophages differ in expression of CD64 [6] and potentially other immunological receptors, including *Tlr7* [9], suggesting differing responsiveness to inflammatory stimuli. Critically, we show the CD11c^+^ subset can be generated and maintained independently of CSF1R signaling. This may have important implications for maintenance of F4/80^lo^MHCII^+^ macrophage function in the face of competition for CSF1 from resident macrophages or inflammatory macrophages recruited during inflammation. For instance, our data may help explain why numbers of CD11c^–^ F4/80^lo^MHCII^+^ macrophages decline relatively rapidly following onset of peritoneal inflammation whereas those of CD11c^+^ cells remain constant throughout [8]. The inference of our findings, that microbiota may regulate the prevalence of CSF1‐independent F4/80^lo^MHCII^+^ macrophages, may be similarly important in the interplay between microbiota and inflammatory diseases of the peritoneal cavity, for example, as may occur during endometriosis [34].

We recently identified a critical role for EGR2 in regulating development of alveolar macrophages including their expression of CD11c [35]. Alveolar macrophages are dependent on GM‐CSF [36], but not on CSF1‐induced expression of EGR2 by monocytes in vitro [35]. EGR2 is also expressed by BM‐derived DC generated with GM‐CSF [37], which largely represents CD11c^+^ monocyte‐derived cells [38]. Furthermore, the microbiota can regulate production of GM‐CSF and prevalence of GM‐CSF‐producing cells [39, 40, 41] and treatment with exogenous GM‐CSF leads to marked expansion of peritoneal CD11c^+^ F4/80^lo^MHCII^+^ macrophages [9]. Surprising then, peritoneal CD11c^+^ F4/80^lo^MHCII^+^ macrophages seemingly persist independently of endogenous GM‐CSF [9]. Although GM‐CSF and CSF1 could play redundant or compensatory roles in regulation of CD11c^+^ F4/80^lo^MHCII^+^ macrophages, a different factor would still be required for induction of CD11c and presumably EGR2 in these cells.

We previously proposed that F4/80^lo^MHCII^+^ macrophages are precursors of resident F4/80^hi^ serous cavity macrophages since they exhibit an overlapping phenotype with resident macrophages of recent monocyte‐origin [6, 15] and can differentiate into F4/80^hi^ MHCII^–^ resident‐like macrophages upon transfer into mice genetically deficient in resident cells [17]. However, this perspective did not account for the possibility that cells of recent monocyte origin could share certain traits irrespective of their dominant macrophage identity and that potential does not necessarily reflect actual fate. Hence, although we found a minor cell‐intrinsic role for EGR2 expression in the differentiation of resident macrophages from the BM in females, the relative lack of reliance of resident macrophages on intrinsic EGR2 compared with the partial and complete reliance of CD11c^–^ and CD11c^+^ F4/80^lo^MHCII^+^ macrophages, respectively, suggests neither population of F4/80^lo^MHCII^+^ macrophages are obligate precursors of monocyte‐derived resident macrophages. Hence, F4/80^lo^MHCII^+^ macrophages appear to largely represent a cul‐de‐sac in the differentiation of monocytes, which reveals an unappreciated level of complexity in monocyte fate determination in this site particularly as it is an essentially fluidic environment [42, 43].

Despite a minor cell‐intrinsic role for EGR2 in generation of resident F4/80^hi^ macrophages in female mice, we found that intact female *Lyz2* ^Cre^.*Egr2* ^fl/fl^ mice had elevated numbers of resident F4/80^hi^ macrophages, suggesting that CD11c^+^ EGR2^+^ F4/80^lo^ peritoneal macrophages may regulate homeostasis of resident F4/80^hi^ cells. If so, this effect must be counteracted by the absence of CD11b^+^ cDC2 and/or CD11c^–^ F4/80^lo^ macrophages since numbers of F4/80^hi^ resident macrophages were normal in female CD11c^Cre^.*Irf4* ^fl/fl^ mice, which lack these cells as well as CD11c^+^ F4/80^lo^ macrophages. Furthermore, any role for CD11c^+^ F4/80^lo^ macrophages in negative regulation of F4/80^hi^ macrophages appears unique to females, as male *Lyz2* ^Cre^.*Egr2* ^fl/fl^ mice exhibited normal numbers of resident macrophages despite loss of CD11c^+^ F4/80^lo^ peritoneal cells. Hence, although we found disruption of microbiota led to elevated numbers of resident F4/80^hi^ macrophages, it seems unlikely this is a direct effect of the loss of CD11c^+^ F4/80^lo^ macrophages that also occurred, since these studies were performed in male mice. What is clear is that biological sex influences many facets of the behavior of macrophages in the peritoneal cavity in nuanced ways.

We also reveal a novel sex and site‐dependent role for RELM‐α in regulating survival and/or differentiation of F4/80^lo^MHCII^+^ macrophages and monocyte‐derived F4/80^hi^ macrophages in the serous cavities. Furthermore, our data suggest this likely results in a reduction in monocyte‐derived TIM4^–^ cells within resident peritoneal F4/80^hi^ macrophages from female RELM‐α‐deficient mice. Hence, our data explain recent observations that, at the population level, resident macrophages from female RELM‐α‐deficient mice express higher levels of *Timd4*, the gene encoding TIM4 [44]. A cell‐intrinsic competitive advantage provided by a largely secreted cytokine like RELM‐α suggests possible autocrine action. Consistent with this, exogenous RELM‐α specifically binds macrophages [45] and can subsequently partner with Bruton's tyrosine kinase [46], a key signaling molecule that prevents activation‐induced apoptosis of macrophages [47]. Alternatively, the functionally related human‐secreted cytokine Resistin relocates to the ER during cell stress, where it provides intrinsic protection from apoptosis [48]. Thus, it is plausible that RELM‐α may mimic this process in mice [48]. Either way, these results reveal that the female peritoneal environment presents a unique survival challenge to monocyte‐derived macrophages, potentially explaining why resident macrophages in this site are more reliant on self‐maintenance [6, 15].

In conclusion, our study sheds light on the diversity of MNPs present in the serous cavities and provides the rationale and relevance for partitioning F4/80^lo^MHCII^+^ macrophages into CD11c^+^ and CD11c^–^ cells. Furthermore, our discovery that cell‐ intrinsic RELM‐α expression contributes to macrophage fitness has potentially broad implications for modeling inflammatory disease in mice, particularly Th2‐biased pathologies wherein alternatively activated RELM‐α^+^ macrophages exhibit an overt competitive advantage [49] and regulate immune pathology [45, 50].

## Materials and methods

### Experimental animals

GM and SPF control C57BL/6J mice, CD11c‐Cre.*Irf4* ^fl/fl^, CD11c‐Cre.*Irf4* ^fl/–^, *Irf4* ^fl/–^, and *Irf4* ^fl/fl^ mice were bred and maintained at the University of Manchester, UK. Effects of antibiotic treatment were studied using C57BL/6J mice bred and maintained under SPF conditions at the University of Glasgow, UK. C57BL/6J CD45.2^+^ and congenic CD45.1^+^CD45.1^–^ and CD45.1^+^CD45.2^+^ mice, *Retnla* ^–/–^ and *Csf1r* ^ΔFIRE/ΔFIRE^ mice, and respective *Retnla* ^+/+^ and *Csf1r* ^+/+^ littermate controls, *Lyz2* ^Cre^.*Rosa26* ^LSL‐CAG‐tdTomato^ *, Fcgr1* ^Cre^.*Rosa26* ^LSL‐RFP/+^, and C57BL/6N mice were bred and maintained under SPF facilities at the University of Edinburgh, UK. *Lyz2* ^Cre^.*Egr2* ^fl/fl^ and *Egr2* ^fl/fl^ mice were bred and maintained at the University of Edinburgh under non‐SPF conditions except where used for generation of BM chimeric animals, for which animals were bred and maintained under SPF conditions. For experiments involving nongenetically altered animals alone, C57BL/6J mice were used unless specified. Identifiers and substrains for the animals used in these studies can be found in Table 1. In some experiments, C57BL/6JCrl mice were purchased from Charles River, UK. All experimental mice were aged match and the sexes used are stipulated in figure legends. Experiments were permitted under license by the UK Home Office and were approved by the University of Edinburgh Animal Welfare and Ethical Review Body, the University of Glasgow Local Ethical Review Panel, or the University of Manchester Animal Welfare and Ethical Review Body. *Fcgr1* ^iCre^ mice are available from Prof. Bernard Malissen under a material transfer agreement with the Centre d'Immunologie de Marseille‐Luminy, Aix Marseille Université.

**Table 1:** List of mouse strains

Strain	Source	Identifier	Background/substrain
C57BL/6J CD45.1^+^	University of Edinburgh		C57BL/6JCrl
C57BL/6J CD45.2^+^	University of Manchester University of Glasgow University of Edinburgh Univeraity of Edinburgh University of Edinburgh		Germfree study – unknown ABX study – C57BL/6JOlaHsd Development post‐birth – C57BL/6JOlaHsd Sex comparison study – C57BL/6JCrl Comparison to C57BL/6N – C57BL/6JCrl
C57BL/6J CD45.1/.2^+^	University of Edinburgh		C57BL/6JCrl
C57BL/6N	University of Edinburgh		C57BL/6NCrl
*Fcgr1* ^Cre/+^.*Rosa26* ^LSL‐RFP/+^	University of Edinburgh	*Fcgr1* ^Cre^ mice [32] *Rosa26* ^LSL‐RFP^ mice [54]	C57BL/6 Substrain undetermined
*Lyz2* ^Cre^ *.Egr2* ^fl/fl^	University of Edinburgh	*Lyz2* ^Cre^ mice [55] *Egr2* ^fl/fl^ mice [56]	C57BL/6 Substrain undetermined
*Lyz2* ^Cre^.*Rosa26* ^LSL‐CAG‐tdTomato^	University of Edinburgh	*Lyz2* ^Cre^ mice [55] *Rosa26* ^LSL‐CAG‐tdTomato^ mice [57]	C57BL/6 Substrain undetermined
CD11c‐Cre.*Irf4* ^fl/fl^	University of Manchester	*Itgax* ^Cre^ mice [58] *Irf4* ^fl/fl^ mice [59]	C57BL/6 Substrain undetermined.
*Csf1r* ^ΔFIRE/ΔFIRE^	University of Edinburgh	[31]	C57BL/6JCrl x CBA
*Retnla* ^–/–^	University of Edinburgh	[50]	C57BL/6JOlaHsd

### Generation and analysis of BM chimeric mice

For generation of mixed BM chimeras, CD45.1^+^CD45.2^+^ mice were exposed to two sequential doses of 5‐Gy γ‐irradiation 1 h apart before being reconstituted immediately with 2–5 × 10^6^ cells of a 50:50 mix of WT (CD45.1^+^) and *Lyz2* ^Cre/+^.*Egr2* ^fl/fl^ or *Egr2* ^fl/fl^ (CD45.2^+^) BM. For generation of tissue‐protected single BM chimeras, CD45.1^+^CD45.2^+^ mice were anaesthetized and then exposed to a single dose of 9.5Gy γ‐irradiation with all but the hind legs being protected by 0.05 m lead shield. The following day, animals were reconstituted with 2–5 × 10^6^ *Retnla* ^–/–^ or *Retnla* ^+/+^ littermate control BM cells. Chimerism was assessed at 8 weeks after reconstitution. For analysis of mixed BM chimeras, residual recipient‐derived CD45.1^+^CD45.2^+^ cells were first excluded. Chimerism of tissue cells was expressed as relative to chimerism of blood monocytes, and in some cases, then subsequently expressed as relative to mean level of chimerism in mice receiving control *Lyz2* ^+/+^.*Egr2* ^fl/fl^ or *Retlna* ^+/+^ BM to account for potential effects of CD45.1 and CD45.2 background.

### Antibiotic treatment

Mice received a cocktail of ampicillin (1 g/L), metronidazole (1 g/L), neomycin (1 g/L), and vancomycin (0.5 g/L) in drinking water for 2 weeks.

### Isolation of cells

Mice were sacrificed by exposure to rising levels of CO_2_ or overdose of anesthetic and death confirmed by cessation of blood flow. The peritoneal cavity and pleural cavities were lavaged with RPMI containing 2 mM EDTA, 1 mM HEPES (Invitrogen) as previously described [51]. In some experiments, blood was taken from the inferior vena cava immediately following peritoneal lavage or from the tail vein prior to necropsy. Blood was immediately mixed in a 10:1 ratio with 0.5 M EDTA. Red blood cells were lysed in blood samples using RBC lysis buffer (BioLegend). Cellular content of lavage samples was determined by cell counting using a Casey TT counter (Roche) together with multicolor flow cytometry.

### Flow cytometry

Flow cytometry and cell sorting adhered to established guidelines [52]. Equal numbers of peritoneal or pleural cells or equal volumes of blood cells were incubated for 10 min at room temperature with Zombie Aqua viability dye (BioLegend) in PBS before incubation with 0.25 μg/mL antibody to CD16/CD32 (BioLegend) in FACS buffer (2 mM EDTA, 0.5% BSA (Sigma), PBS. Cells were incubated with a combination of the following antibodies in FACS buffer on ice for 30 min: CD226 (10E5), F4/80 (BM8), Ly6C (HK1.4), CD11b (M1/70), MHCII (M5/114.15.2), CD19 (6D5), CD3 (17A2), CD11c (N418), CSF1R (AFS98), CD45.1 (A20), CD45.2 (104), Ly6G (1A8), CD102 (3C4), TIM4 (RMT4‐54), and Siglec‐F (ES22‐10D8; Miltenyi Biotec). Antibodies were from BioLegend unless otherwise stated. Cells were then washed with FACS buffer and, where required, were stained with fluorochrome‐conjugated streptavidin (BioLegend). In some experiments, cells were fixed using the Foxp3 staining buffer kit (eBioscience) according to the manufacturers protocol, followed by intracellular staining with antibodies to IRF4 (IRF4.3E4; BioLegend), or EGR2 (Erongr2; Invitrogen), or purified, or biotinylated polyclonal rabbit antibody to RELM‐α (Peprotech) followed by Zenon anti‐rabbit reagent (Invitrogen) or streptavidin‐conjugated fluorochromes. Samples were acquired using FACS LSRFortessa (BD) and analyzed using Flowjo (version 9.9.6, Treestar). Doublets and dead cells were excluded from analysis using forward scatter area versus height and positivity for Zombie Aqua dye, respectively, and T cells, B cells, eosinophils, and neutrophils excluded using a Lineage^–^ gate comprising antibodies to CD3, CD19, Siglec‐F, and Ly6G.

### RNA sequencing and analysis of microarray data

Microarray data of different myeloid populations produced by the ImmGen consortium (GEO15907) was downloaded from Gene Expression Omnibus and differential gene expression determined as previously detailed [6]. Differentially expressed genes were determined using a minimum of a twofold gene expression and adjusted *p*‐value of 0.01 (*t*‐test) on log2 transformed data. Details of the sorting strategies used to isolate each population can be found at http://www.immgen.org. Heatmaps display the normalized log2 expression.

For RNAseq, CSF1R^+^ MHCII^+^ Ly6C^–^ CD11b^+^ CD102^–^ Lin^–^ (F4/80^lo^) and CD11b^+^ Lin^–^ CD102^+^ (F4/80^hi^)_macrophages were FACS‐purified from the peritoneal cavities of unmanipulated male and female mice, as previously detailed [15]. Briefly, 25 000 cells of each population were sorted into 500 μL of RLT buffer (Qiagen) and snap‐frozen on dry ice. The RNeasy Plus Micro Kit (Qiagen) was used to isolate RNA, at which point triplicates of 25 000 cells for each population were pooled. Ten nanograms of total RNA was amplified and converted to complementary DNA using Ovation RNA‐Seq System V2 (Nugen). Sequencing was performed by Edinburgh Genomics using the Illumina HiSeq 4000 system (75PE). Raw map reads were processed with the R package DESeq2 [53] to generate the DEGs, and the normalized count reads to generate and visualize on heatmaps generated by the R package pheatmap. Samples with >5% of reads mapped to ribosomal RNA were removed from analysis. DEGs were determined using at least a 1.5‐fold difference and adjusted *p* < 0.01 for each comparison. Data for F4/80^hi^ CD102^+^ macrophages are published [15] and deposited in National Center for Biotechnology Information Gene Expression Omnibus public database (www.ncbi.nlm.nih.gov/geo/) with the code 149014 (F4/80^hi^ CD102^+^ macrophages). Data for CSF1R^+^ MHCII^+^ F4/80^lo^ macrophages can be accessed with the code GSE200630.

### Statistics

Data were analyzed using Prism 6/7 (Graphpad Software). Where required, data were log transformed to achieve equal variance and normal distribution before testing with one‐way ANOVA, followed by Tukey's multiple comparison test or Student's *t*‐test followed by Holm–Sidak correction, if required. Grubbs test identified a single outlier in the *Egr2* ^fl/fl^ control group from experiments presented in Figure 3 and Supporting information Figure 5, which was removed prior to analysis and generation of figures. One 50:50 mixed BM chimera was excluded due to poor depletion of endogenous BM, as evidenced by >85% of monocytes of host origin. All other 50:50 mixed BM chimeras exhibited fewer than 10% residual host‐derived cells.

## Author contributions

DR and PAL designed, performed, analyzed, and interpreted experiments. NJS analyzed microarray and RNA‐sequencing data; ABB and LMH performed experiments; CP provided mice. SWFM and ASM supplied mice and reagents, and designed experiments. CCB and SJJ designed, performed, analyzed, and interpreted experiments, and wrote the manuscript. SJJ conceived and supervised the project.

## Conflict of interest

Authors have no conflict of interest.

## Ethics approval

Animal experiments were permitted under license by the UK Home Office (70/8218, PP0860257, P64BCA712 and 70/7815), and were approved by the University of Edinburgh Animal Welfare and Ethical Review Body, the University of Glasgow Local Ethical Review Panel, or the University of Manchester Animal Welfare and Ethical Review Body.

### Peer review

The peer review history for this article is available at https://publons.com/publon/10.1002/eji.202149756

**Abbreviations**
cDC: conventional DC
EGR2: early growth response 2
FIRE: Fms‐intronic regulatory element
GF: germ‐free mice
MNPs: mononuclear phagocytes
RFP: red fluorescent protein
SPF: specific‐pathogen‐free

## Supporting information



Table S1

Table S2

Supporting Information

## Data Availability

RNA‐seq data supporting the findings of this study have been deposited in the National Center for Biotechnology Information Gene Expression Omnibus public database (www.ncbi.nlm. nih.gov/geo/) under accession code [GSE200630]. Other data will be made available from the corresponding author upon reasonable request.
