# Supplementary material for: CD11c identifies microbiota and EGR2‐dependent MHCII+ serous cavity macrophages with sexually dimorphic fate in mice
Source: Eur J Immunol. 2022 May 24;52(8):1243–57. doi: 10.1002/eji.202149756 (PMC7613339; doi:10.1002/eji.202149756)
Supplement: Supplementary file 3 — Supporting Information [file EJI-52-1243-s001.pdf]

# SUPPORTING INFORMATION

## CD11c identifies microbiota and EGR2-dependent MHCII$^+$ serous cavity macrophages with sexually dimorphic fate in mice.

Calum C. Bain, Pieter A. Louwe, Nicholas J. Steers, Alberto Bravo-Blas, Lizi M. Hegarty, Clare Pridans, Simon W.F. Milling, Andrew S. MacDonald, Dominik Rückerl, Stephen J. Jenkins

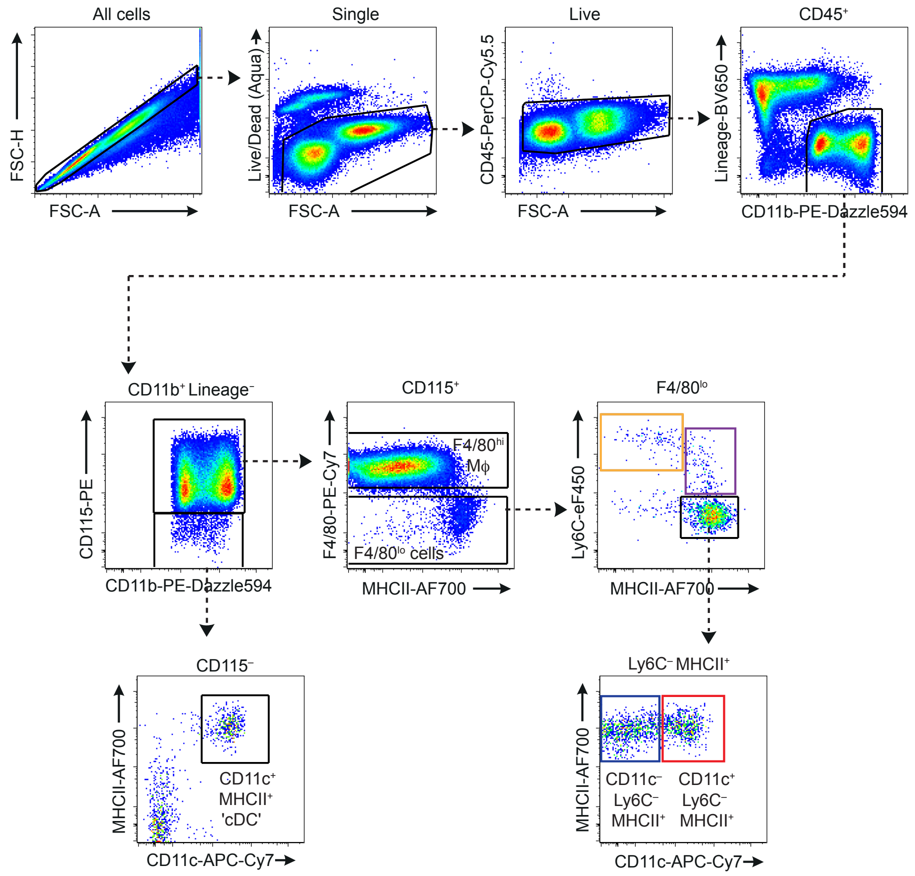


**Supplementary Fig. 1:**
Gating strategy used to identify CD11b$^+$ DC and F4/80$^{hi}$ and F4/80$^{lo}$ subsets of CD11b$^+$ CD115$^+$ CD45$^+$ Lin$^-$ mononuclear phagocytes obtained from the peritoneal cavity of unmanipulated C57BL/6 mice.

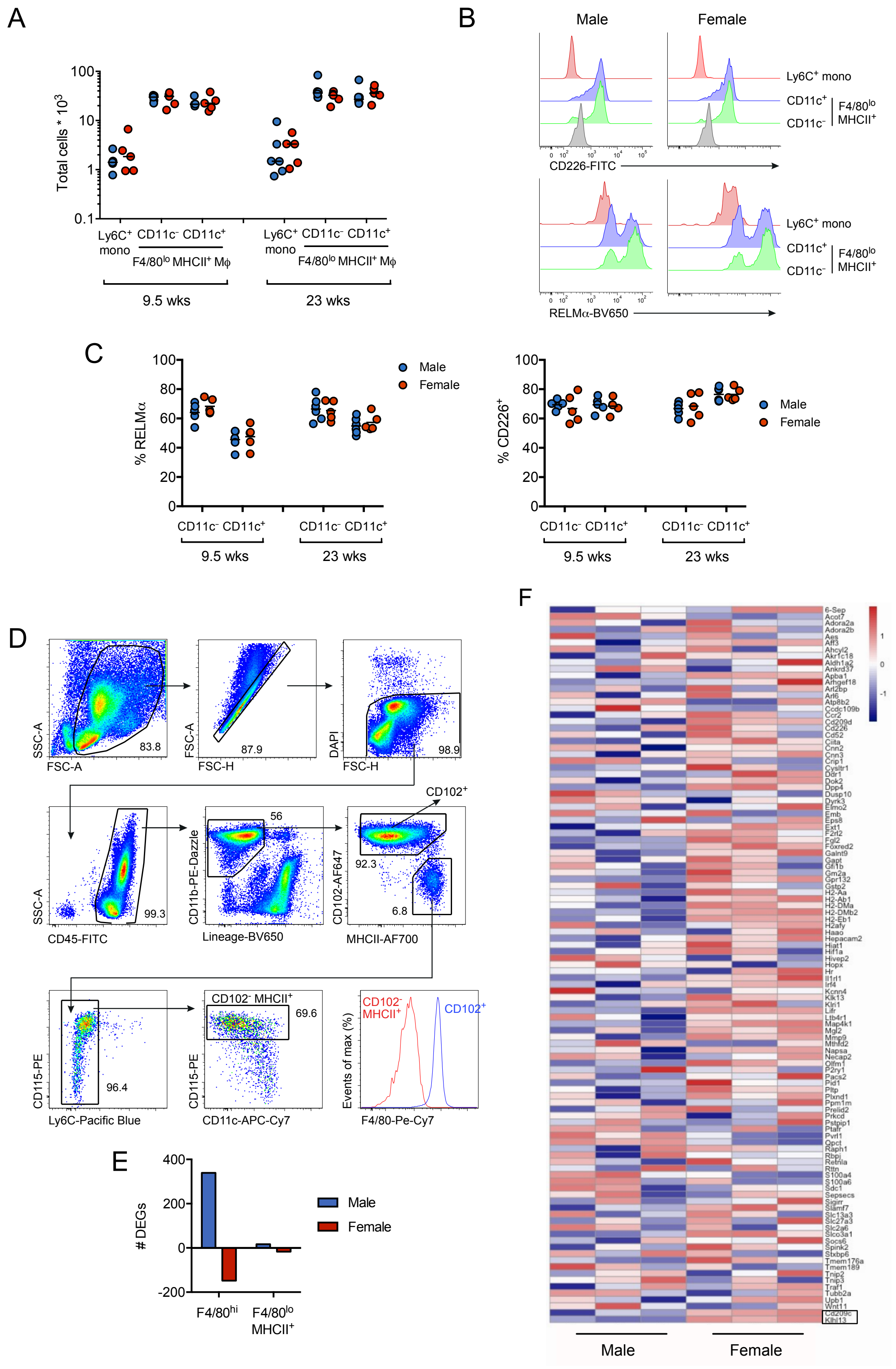


**Supplementary Fig. 2:**

**A**. Absolute number of Ly6C$^{+}$ monocytes and CD11c$^{-}$ and CD11c$^{+}$ fractions of Ly6C$^{-}$ MHCII$^{+}$ F4/80$^{lo}$ macrophages in Lin$^{-}$ CD11b$^{+}$ CD115$^{+}$ peritoneal leukocytes obtained from 9.5 and 23 week old male and female C57BL/6 mice.

**B**. Representative expression of intracellular RELM$\alpha$ and surface CD226 by Ly6C$^+$ monocytes and CD11c$^-$ and CD11c$^+$ fractions of Ly6C$^-$ MHCII$^+$ F4/80$^{lo}$ macrophages in Lin$^-$ CD11b$^+$ CD115$^+$ peritoneal leukocytes obtained from male and female mice in (A). Shaded histograms represent FMO control.

**C**. Frequency of RELM$\alpha^+$ and CD226$^+$ cells within CD11c$^-$ and CD11c$^+$ fractions of Ly6C$^-$ MHCII$^+$CD115$^+$F4/80$^{lo}$ cells from the peritoneal cavity of male and female mice detailed in (A).
(A, C) Symbols represent individual mice with 5-6 mice per group pooled from two independent experiments.

**D**. Gating strategy for the purification of CD102$^+$ and CD102$^-$ MHCII$^+$ macrophages for population level RNAseq, and overlay showing expression of F4/80 by CD102-defined macrophages. Data for CD102$^+$F4/80$^{hi}$ macrophages published previously(15).

**E.** The number of genes expressed more highly ($p<0.01$, >1.5 fold change) by male or female CD102$^+$ (F4/80$^{hi}$) or CD102$^-$ MHCII$^+$ (F4/80$^{lo}$) macrophages FACs purified from the cavity of 10-12 week old mice. Differently expressed genes were determined using 3 samples per group, with both macrophage populations purified from the same animals. Data for CD102$^+$F4/80$^{hi}$ macrophages published previously(15).

**F**. Gene expression profile of the 112 genes identified by Kim et al.(7) to distinguish F4/80$^{lo}$ MHCII$^+$ peritoneal macrophages from F4/80$^{hi}$ peritoneal macrophages and resident macrophages in the lung, brain and spleen. Differentially expressed genes are highlighted.

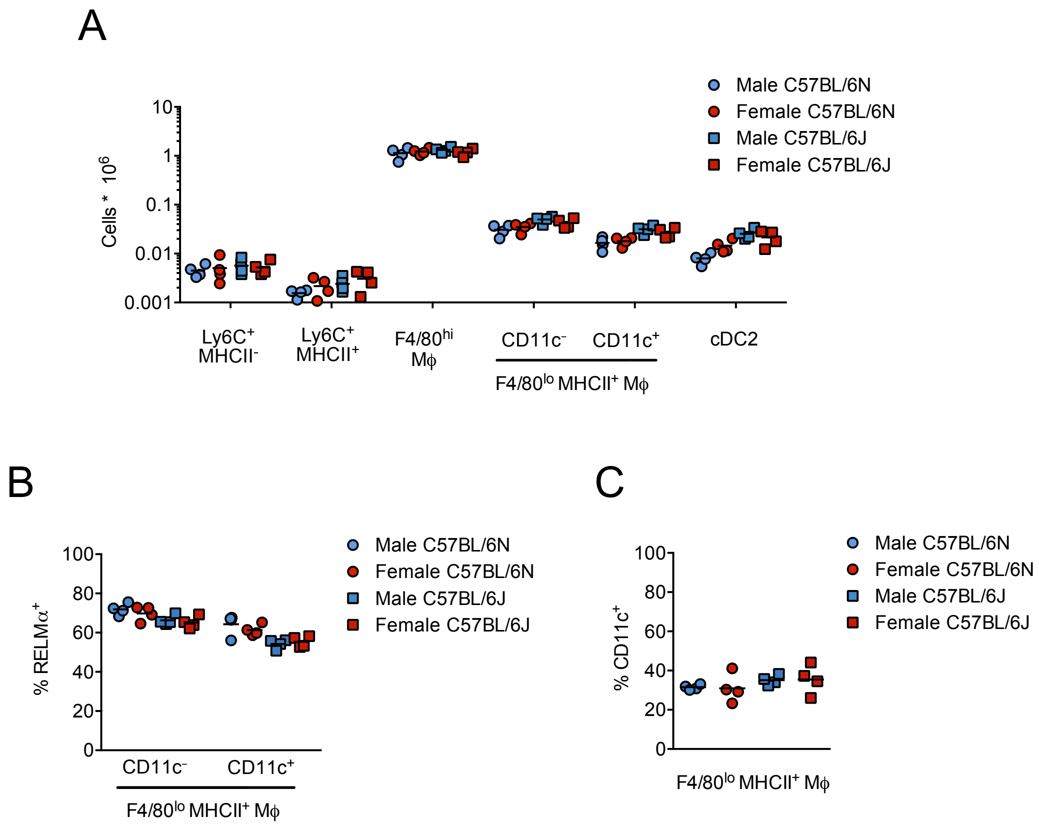


**Supplementary Fig. 3:**

**A**. Absolute number of MHCII$^-$ and MHCII$^+$ Ly6C$^+$ monocytes, F4/80$^{hi}$ resident macrophages and CD11c$^-$ and CD11c$^+$ fractions of Ly6C$^-$ MHCII$^+$ F4/80$^{lo}$ macrophages in Lin$^-$ CD11b$^+$ CD115$^+$ peritoneal leukocytes and Lin$^-$ CD11c$^+$ MHCII$^+$ CD11b$^+$ CD115$^-$ cDC2 in obtained from age-matched male and female C57BL/6J and C57BL/6N mice.

**B**. Frequency of RELM$\alpha^+$ cells within CD11c$^-$ and CD11c$^+$ fractions of Ly6C$^-$MHCII$^+$CD115$^+$F4/80$^{lo}$ cells from the peritoneal cavity of male and female mice detailed in (A).

**C**. Proportion of CD11c$^+$ cells within Ly6C$^-$MHCII$^+$CD115$^+$F4/80$^{lo}$ cells from the peritoneal cavity of male and female mice detailed in (A).

(A-C) Symbols represent individual mice with 4 mice per group pooled from two independent experiments.

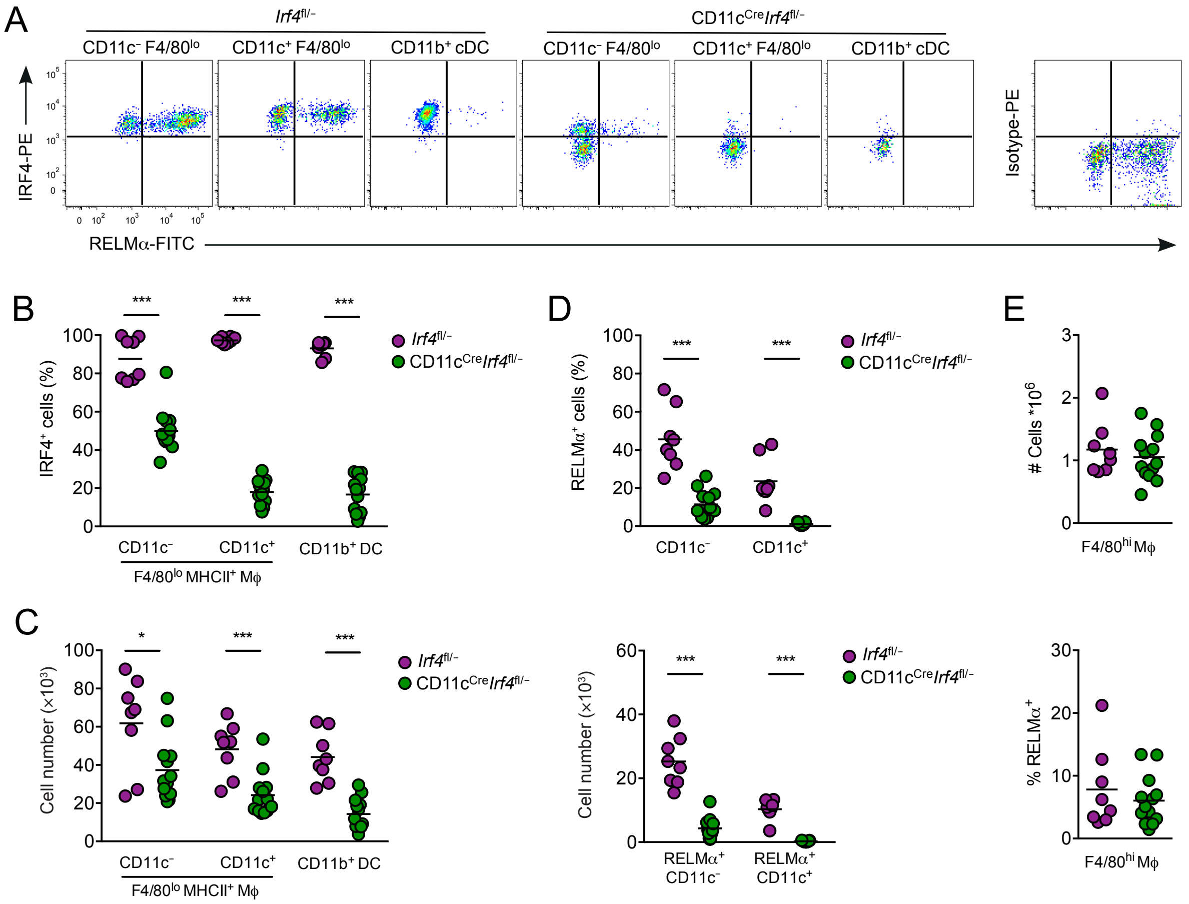


**Supplementary Fig. 4:**

**A**. Representative expression RELMα and IRF4 by CD11c-defined subsets of F4/80$^{lo}$ MHCII$^{+}$ CD115$^{+}$ peritoneal macrophages and CD11b$^{+}$ cDC from age-matched female *Irf4*$^{fl/fl}$ and *Irf4*$^{fl/-}$ (Cre$^{-}$) or *Cd11c*$^{Cre}$*Irf4*$^{fl/fl}$ and *Cd11c*$^{Cre}$*Irf4*$^{fl/-}$ (Cre$^{+}$) mice. Isotype control for IRF4 performed on all MHCII$^{+}$ F4/80$^{lo}$ macrophages combined.

**B**. Frequency of IRF4$^{+}$ cells within CD11c-defined subsets of F4/80$^{lo}$ MHCII$^{+}$ CD115$^{+}$ peritoneal macrophages and CD11b$^{+}$ cDC from Cre$^{-}$ and Cre$^{+}$ mice in (A).

**C**. Absolute number of CD11c-defined subsets of F4/80$^{lo}$ MHCII$^{+}$ CD115$^{+}$ peritoneal macrophages and CD11b$^{+}$ cDC from Cre$^{-}$ and Cre$^{+}$ mice in (A).

**D**. Frequency (top) and absolute number (bottom) of RELMα$^{+}$ cells within CD11c-defined subsets of F4/80$^{lo}$ MHCII$^{+}$ CD115$^{+}$ peritoneal macrophages from Cre$^{-}$ and Cre$^{+}$ mice in (A).

**E**. Absolute number (top) and proportion that express RELMα (bottom) of F4/80$^{hi}$ resident peritoneal macrophages from mice in (A).

(B-E) Symbols represent individual mice with 8 (Cre$^{-}$) and 13 (Cre$^{+}$) mice per group pooled from two independent experiments. *$p<0.05$, ***$p<0.001$ (Student's *t*-test with Holm-Sidak correction).

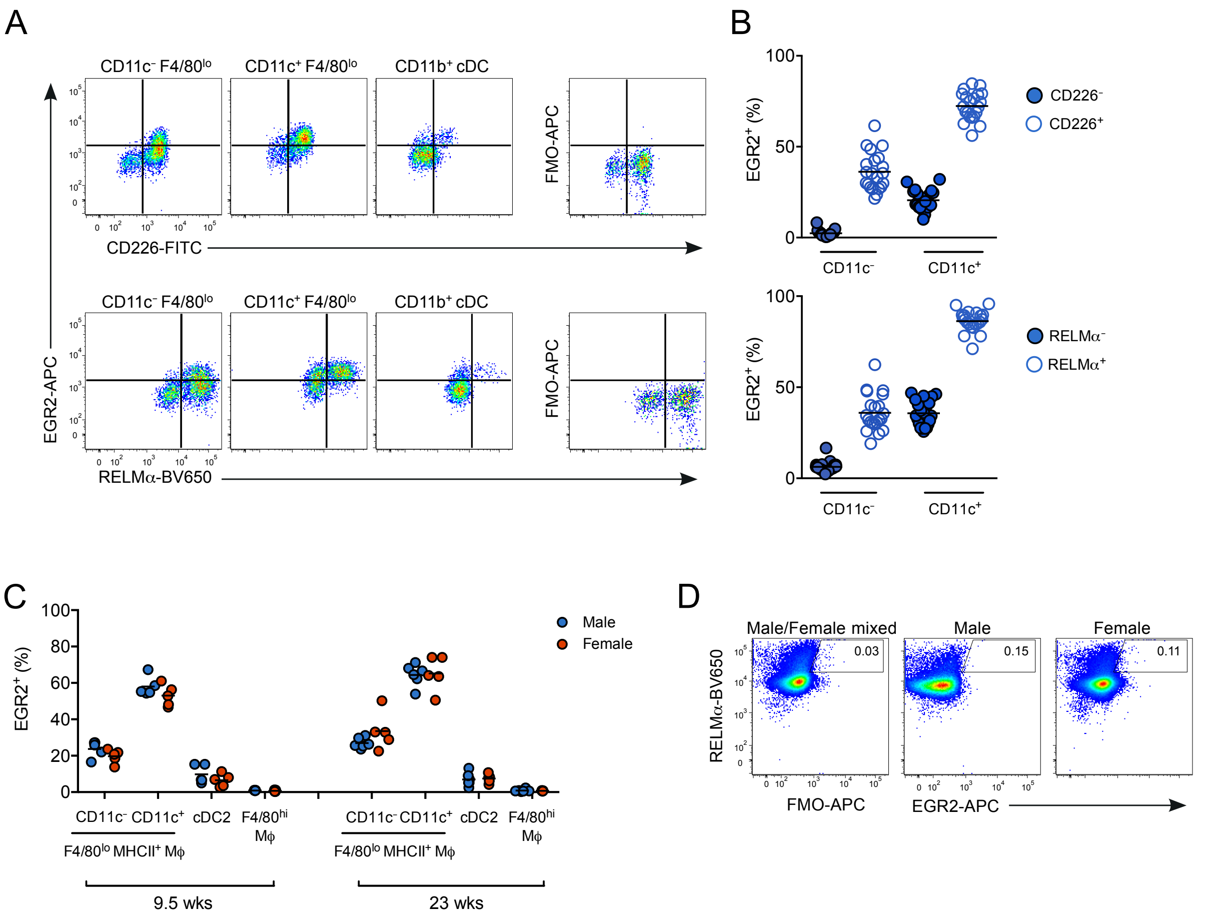


**Supplementary Fig. 5:**

**A**. Representative expression of RELMα and EGR2 by CD11c-defined subsets of F4/80$^{lo}$ MHCII$^{+}$ CD115$^{+}$ macrophages and CD11b$^{+}$ cDC from the peritoneal cavity of 9.5 and 23 week old male and female C57BL/6 mice. FMO control for EGR performed on all MHCII$^{+}$F4/80$^{lo}$ macrophages combined.

**B**. Frequency of EGR2$^{+}$ cells within CD11c and CD226 (top graph) or RELMα$^{+}$ (bottom graph) -defined subsets of F4/80$^{lo}$MHCII$^{+}$CD115$^{+}$ peritoneal macrophages pooled from 21 mice in (A).

**C**. Frequency of EGR2$^{+}$ cells within CD11c-defined subsets of F4/80$^{lo}$ MHCII$^{+}$ CD115$^{+}$ macrophages, CD11b$^{+}$ cDC and F4/80$^{hi}$ resident macrophages from the peritoneal cavity of 9.5 and 23 week old male and female C57BL/6 mice from (A). Symbols represent individual mice with 5 (9.5 week old males and females, and 23 wk old females) and 6 (23 wk old males) mice per group pooled from two independent experiments.

**D**. Representative expression of RELMα and EGR2 by F4/80$^{hi}$ resident peritoneal macrophages from a single male and female mouse in (A). FMO control for EGR performed on mixed cells from male and female mice.

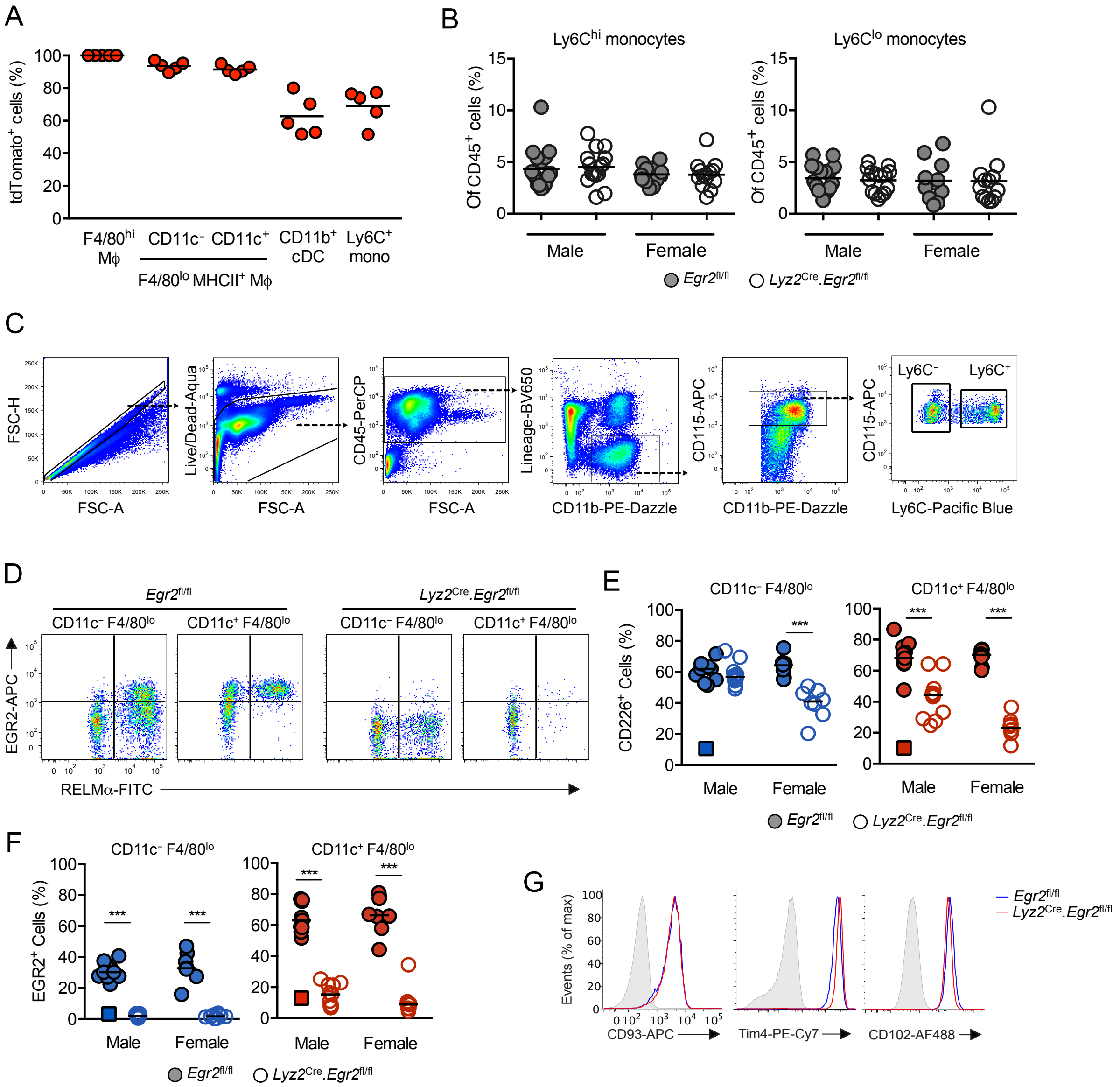


**Supplementary Fig. 6:**

**A**. Frequency of tdTomato$^+$ cells within CD11c-defined subsets of F4/80$^{lo}$ MHCII$^+$ CD115$^+$ macrophages, CD11b$^+$ cDC and F4/80$^{hi}$ resident macrophages from the peritoneal cavity of *Lyz2*$^{Cre}$.*Rosa26*$^{LSL\text{-}Ai14}$ mice. Symbols represent 5 individual mice pooled from two independent experiments.

**B.** Frequency of Ly6C$^+$ (left) and Ly6C$^-$ (right) monocytes of live CD45$^+$ blood leukocytes from age-matched male and female *Lyz2*$^{Cre}$.*Egr2*$^{fl/fl}$ (Cre$^+$) or *Egr2*$^{fl/fl}$ (Cre$^-$) mice. Symbols represent individual mice pooled from three independent experiment, with 13 (female Cre$^+$ and Cre$^-$), 15 (male Cre$^-$) and 17 (male Cre$^-$) mice per group.

**C**. Gating strategy showing identification of blood Ly6C$^+$ and Ly6C$^-$ monocytes from mice in (B).

**D**. Representative expression of RELMα and EGR2 by CD11c-defined subsets of F4/80$^{lo}$ MHCII$^+$ CD115$^+$ macrophages from age-matched male and female *Lyz2*$^{Cre}$.*Egr2*$^{fl/fl}$ (Cre$^+$) or *Egr2*$^{fl/fl}$ (Cre$^-$) mice.

**E**. Frequency of EGR2-expressing cells within CD11c$^-$ (left) and CD11c$^+$ (right) subsets of F4/80$^{lo}$ MHCII$^+$ CD115$^+$ macrophages of mice in (D). Symbols represent individual mice pooled from two

independent experiment, with 8 (female $Cre^+$ and $Cre^-$) or 11 (male $Cre^-$ and $Cre^-$) mice per group. ***p<0.001 (Student's *t*-test with Holm-Sidak correction).

**F**. Frequency of $CD226^+$ cells within $CD11c^-$ (left) and $CD11c^+$ (right) fractions of $Ly6C^-$ $MHCII^+CD115^+F4/80^{lo}$ cells from the peritoneal cavity of $Cre^+$ and $Cre^-$ mice in (E). ***p<0.001 (Student's *t*-test with Holm-Sidak correction).

**G**. Representative histograms showing expression of CD93 and CD102 by $F4/80^{hi}$ peritoneal macrophages from female *Lyz2*$^{Cre}$.*Egr2*$^{fl/fl}$ ($Cre^+$) or *Egr2*$^{fl/fl}$ ($Cre^-$) mice.

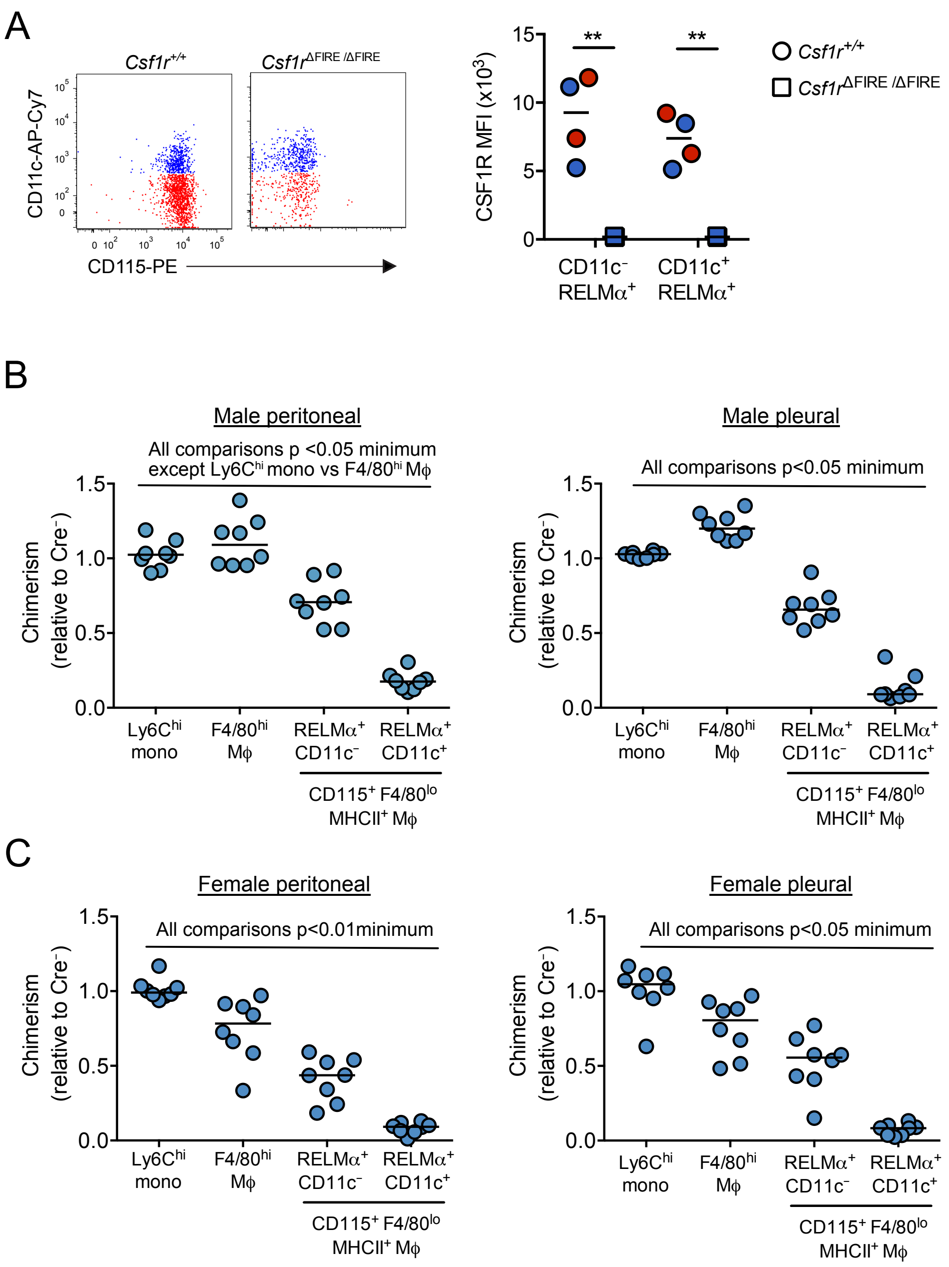


**Supplementary Fig. 7:**

**A**. Representative expression of CD115 (CSF1R) and CD11c on all RELMα$^{+}$ F4/80$^{lo}$MHCII$^{+}$ macrophages from *Csf1r*$^{+/+}$ and *Csf1r*$^{\Delta FIRE/\Delta FIRE}$ mice and mean fluorescence intensity of CD115 staining on CD11c-defined subsets of these cells. Symbols represent individual male (blue) or female (red) animals, with 4 *Csf1r*$^{+/+}$ and 3 *Csf1r*$^{\Delta FIRE/\Delta FIRE}$ mice pooled from two independent experiments. **p<0.01 (Student's *t*-test with Holm-Sidak correction).

**B**. Contribution of CD45.1$^{-}$ CD45.2$^{+}$ *Egr2*$^{fl/fl}$ bone marrow to the indicated peritoneal and pleural cavity populations in male mixed chimeras given Cre$^{+}$ bone marrow. Chimerism was normalized to Ly6C$^{hi}$ blood monocytes before normalization to chimerism in mice given Cre$^{-}$ bone marrow. Data taken from **Fig. 4F**, and represent 8 mice per group pooled from 2 independent experiments. Data analysed by One-way ANOVA with Tukey's test for multiple comparisons.

**C**. As in (B) but for female bone marrow chimeric mice. Data taken from **Fig. 4G**, and represent 8 mice per group pooled from 2 independent experiments. Data analysed by One-way ANOVA with Tukey's test for multiple comparisons.

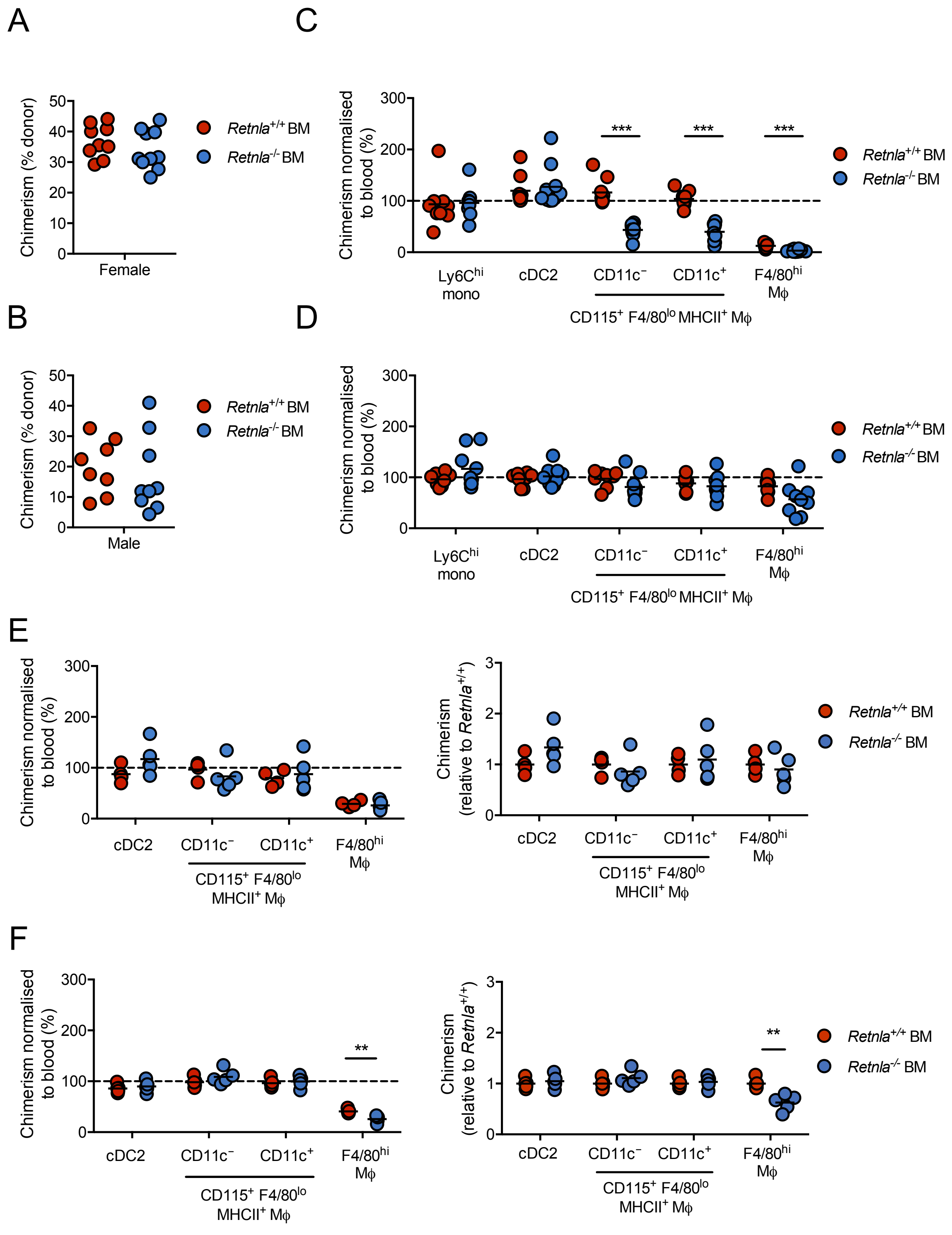


**Supplementary Fig. 8:**

**A**. Contribution of CD45.2$^{+}$ CD45.1$^{-}$ donor-derived cells within blood Ly6C$^{hi}$ monocytes of female tissue-protected CD45.1$^{+}$CD45.1$^{+}$ mice given *Retnla*$^{+/+}$ or *Retnla*$^{-/-}$ bone marrow. Data represent 9 (*Retnla*$^{+/+}$) or 10 (*Retnla*$^{-/-}$) mice per group pooled from 2 independent experiments.

**B**. As (A) but for male chimeras. Data represent 8 (*Retnla*$^{+/+}$) or 9 (*Retnla*$^{-/-}$) mice per group pooled from 2 independent experiments.

**C**. Contribution of CD45.1$^{-}$ CD45.2$^{+}$ bone marrow to the indicated peritoneal populations in female tissue-protected chimeras from (A) normalized to blood Ly6C$^{hi}$ monocytes. ***$p<0.001$ (Student's *t*-test with Holm-Sidak correction).

**D**. As (C) but for male tissue-protected chimeras.

**E**. Contribution of CD45.1$^{-}$ CD45.2$^{+}$ bone marrow to the indicated pleural populations in male tissue-protected chimeras from (A) normalized to blood Ly6C$^{hi}$ monocytes (left graph) before normalization to chimerism in mice receiving *Retnla*$^{+/+}$ bone marrow (right graph). Data represent 4 (*Retnla*$^{+/+}$) or 5 (*Retnla*$^{-/-}$) mice per group from 1 experiment.

**F**. As (E) but for pleural populations from female tissue-protected chimeras. Data represent 5 (*Retnla*$^{+/+}$) or 5 (*Retnla*$^{-/-}$) mice per group from 1 experiment. **$p<0.01$ (Student's *t*-test with Holm-Sidak correction).
